# Innovative Rehabilitation of an Anterior Cruciate Ligament Tear in a Football Player: Muscle Chain Approach—A Case Study

**DOI:** 10.3390/jcm14144983

**Published:** 2025-07-14

**Authors:** Pablo Ortega-Prados, Manuel González-Sánchez, Alejandro Galán-Mercant

**Affiliations:** 1 Department of Physiotherapy, University of Malaga, 29071 Málaga, Spain; pablortega91@hotmail.com (P.O.-P.); mgsa23@uma.es (M.G.-S.); 2 Institute of Biomedical Research of Malaga (IBIMA), 29010 Malaga, Spain; 3 MOVE-IT Research Group, Department of Nursing and Physiotherapy, Faculty of Nursing and Physiotherapy, University of Cádiz, 11003 Cádiz, Spain; 4 Biomedical Research and Innovation Institute of Cádiz (INiBICA) Research Unit, Puerta del Mar University Hospital (Cádiz), University of Cádiz, 11002 Cádiz, Spain

**Keywords:** anterior cruciate ligament, functional rehabilitation, football, sports injuries, muscle chains

## Abstract

**Background:** The incidence of anterior cruciate ligament ruptures in football has experienced a marked increase in recent years, affecting both professional and amateur players. This injury is characterised by being highly disabling, causing the player to withdraw from the field of play for prolonged periods and there is no clear consensus on how to carry out the different phases of rehabilitation, which poses a major challenge for health professionals. **Case presentation:** This study followed a semi-professional player who suffered an anterior cruciate ligament tear following two forced valgus actions without direct contact in the same match. **Outcome and follow-up:** The patient underwent surgery using an autologous hamstring graft. He followed a progressive rehabilitation programme consisting of one preoperative phase and six phases after the operation. After a 12-month follow-up, with exercises aimed at perfecting step-by-step basic and specific physical skills, the player showed a complete functional recovery, achieving the desired parameters. **Conclusions:** This case highlights the importance of structured rehabilitation adapted to the specific needs of the football player through an approach with coherent progressions, which considers the muscle chains that determine the movements performed on the football pitch.

## 1. Background

Anterior cruciate ligament (ACL) rupture is a very common injury in sports such as gymnastics, skiing or team sports, especially football, and leads to about 100,000–200,000 injuries per year [1,2,3]. It is an increasingly common injury for both professional and amateur players [2,3,4]. With a higher risk in competition [5], which can be up to 14 times higher in professional leagues compared to training sessions [4].

The mechanism of injury can vary depending on the situation, with 36% of ACL tears being direct or indirect contact [6]. It is usually a deceleration action, jumping/landing or cutting the ball without contact, and often involves a change of direction [1,2,3,7,8].

The foot is fixed in the grass and a valgus movement of the knee occurs (with little flexion), involving external rotation of the femur and internal rotation of the tibia, which together with the axial load of body weight, can lead to an anterior drawer or subluxation (exacerbated by quadriceps forces and patellar tendon angle) and a posterolateral bony haematoma of the tibia [9,10].

In addition, this action may be complemented by a flattening of the foot, which rotates externally and involves abduction of the hip and ipsilateral trunk tilt and contralateral rotation [2]. It should be noted that most ACL tears occur in an action where the knee has a flexion of less than 20° and the hip does not exceed a flexion of 40° [6].

Some authors propose to reduce the number of ACL injuries through knowledge of the mechanism of injury and correct preventive work [2,3,6]. For players already suffering from this injury, the rehabilitation process is crucial for optimal recovery and to avoid these mechanisms when competing [2], as only 65% return to competition with the same pre-injury fitness levels as before the injury [11] and relapse rates vary between 1.5% and 37.5% [12,13]. However, there is one side that advocates accelerated rehabilitation, which involves competing after approximately 6 months, and another, more conservative option, which involves 9–12 months [11,14,15,16,17,18] and which takes into account the type of surgery and the maturation process of the plasty [11,19,20,21].

In this sense, a good intervention strategy in the preoperative, postoperative and return to training phase will help to avoid complications and improve neuromuscular control, range of motion and above all, with a view to returning to competition, the ability to cope with possible disturbances and imbalances together with improving the coordination and quality of key movements such as running technique, braking, changes of direction, jumps and landings, striking, etc. [2,3,4,22,23,24,25,26].

The purpose of this case study is to propose an exercise-based treatment protocol for football players undergoing ACL hamstring surgery that divides recovery from injury into different phases until return to competition. It provides an example framework to help professionals involved in the rehabilitation process to enrich and improve their therapies in each phase, achieving coherent objectives in a safe and effective way.

## 2. Case Presentation

- Medical history:

A semi-professional football player, 29 years old, breaks his ACL after two actions. In the first action, the player goes to press and steal the ball. He makes a decelerative gesture through a first contact with the heel on the grass (poor load absorption); this causes his knee to go into valgus, while he loses stability in his hip. After this, the player decides to continue playing with discomfort, receives an aerial ball, prepares to make a pass, leaves his weight on the right leg while arming the left leg; at the moment in which the left leg passes to make the concentric gesture of the strike, the player loses stability, notices something similar to a knee subluxation (anterior drawer) and falls to the grass after hitting the ball.

In both actions the player heard a ‘pop’ in the right knee that made him collapse to the ground. He felt that the knee could dislocate, and he could not bear weight on his right side.

- Examination and evaluation:

The day after the injury, a physiotherapy assessment was carried out (Table 1), the results of which led to a severe injury.

Eight days later, a Magnetic Resonance Imaging (MRI) was performed [31]. The MRI revealed a complete ACL tear, longitudinal tear of the posterior horn of the internal meniscus and radial tear of the body of the external meniscus. Two weeks and three days after the injury, physiotherapy sessions were started. Nine physiotherapy sessions were performed, and surgery was subsequently performed.

The patient provided informed consent for publication of this case report and accompanying images. The rehabilitation protocol was approved by University of Málaga Ethics Committee, with approval number 117/2025.

## 3. Treatment

The decision was made to undergo surgical treatment given the level of sport the individual was involved in. The following surgery was carried out:

1. ACL ligamentoplasty with autologous hamstring graft. Toggleloc + Screw fixation [21,32].
2. Partial meniscectomy of the internal meniscus (posterior horn) and a regularisation of the free edge of the external meniscus [33,34].

The player was discharged with his knee immobilised in full extension. The intervention carried out in the different phases of the player’s rehabilitation process is detailed below.

### 3.1. Phase 0: Preoperatory (2.5 Weeks)

The objectives for this phase were to assess the patient’s initial condition and its improvement after the process, eliminate pain and swelling, improve range of motion (ROM) and increase the muscle mass and strength-generating capacity of both lower limbs (LL) avoiding asymmetries between them, thus restoring proper knee function [22,35].

- First week objectives: Pain equal to or less than 3/10 on the Visual Analogue Scale (VAS), decrease swelling between 0 and 1+ [36], and try not to have a large loss of muscle mass (>4.5 cm circumference of the distal thigh) [37,38] or muscle recruitment.

In these sessions, manual and radiofrequency treatments (TCare Compact XP equipment, PRIM, Madrid, Spain), also known as diathermy or tecartherapy, were carried out on a stretcher [39] and above all, a preoperative exercise-based approach was used to safely achieve gains in strength and hypertrophy.

In these cases, the risk of meniscal reinjury is associated with inadequate anteroposterior and rotational control [40,41,42]. Since the ACL tear was complete and knee stabilisation was mainly mediated by the hamstring musculature, exercises with impact and/or rotations were eliminated [40,41,42].

**Figure 1:**
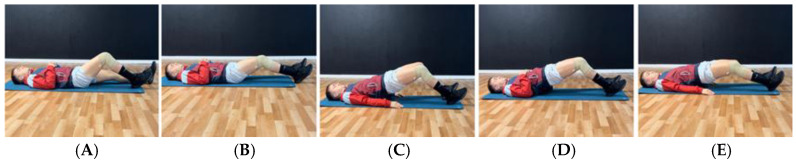
Example of exercise progression to reduce symptomatology. (**A**): Isometric push with heel; (**B**): bilateral isometric push with heels in long lever; (**C**): isometric bridge with the help of arms; (**D**): bridge using elbows; (**E**): isometric bridge with greater activation of the hamstrings in long lever (less involvement of the gluteus muscles).

The main strategy was to start with isometric contractions to reduce pain [43] and to give confidence to the patient. Figure 1 shows a progression from the beginning of the first session, where the subject pushes heels against the floor to activate the posterior chain, then performs the same movement with a longer lever and then performs a gluteal bridge with the help of arms, then without help and finally with a long lever and greater activation of the hamstrings.

**Figure 2:**
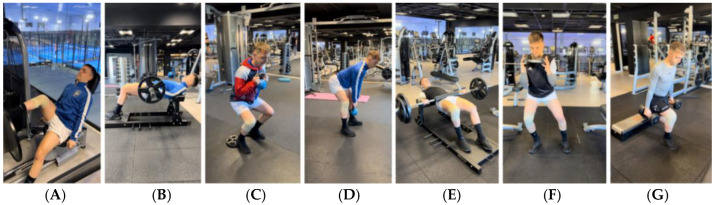
Example of exercise progression to decrease symptomatology and maintain strength/muscle mass levels. (**A**): Unilateral isometric push press; (**B**): bilateral isometric hip thrust; (**C**): isometric squat with wedge; (**D**): isometric deadlift; (**E**): asymmetric isometric hip thrust; (**F**): isometric squat with weight shift; (**G**): Bulgarian squat in isometry.

Difficulty was added according to the player’s tolerance. Because the player had 5/10 VAS pain, fear and a lot of stiffness, isometric exercises were chosen in the activation phase of the first sessions (Figure 2). Isometric exercises were performed, starting with bilateral short lever and closed kinetic chain (CKC) to avoid a possible anterior tibial translation [40,41,42,44,45], progressing to exercises in asymmetric positions with longer or even unilateral levers of movement.

- Objectives of the second week: Gain mobility (full extension and flexion) and progress with the loads (increase weight and symmetry LL > 90°).

**Figure 3:**
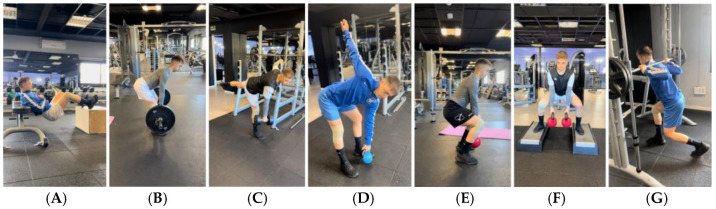
Example of exercise progression to gain mobility and progress through the loads. (**A**): Elevated hip thrust; (**B**): Romanian deadlift; (**C**): unilateral deadlift with stability; (**D**): deadlift with trunk rotation; (**E**): squat; (**F**): sumo squat; (**G**): lunge.

As the player’s ROM, contraction capacity, motor control, sense of stability, etc., improved (Table 2), the load, volume and intensity were increased and more dynamism (controlled concentric and eccentric contractions) was added. In summary, basic exercises such as gluteal or hamstring bridges, deadlifts, hip thrusts, squats and lunges were chosen, and all kinds of variations were applied in a safe, coherent and progressive way to enrich each exercise with different stimuli, planes, etc. (Figure 3).

- Third week objective: Maintain functionality, gait without compensations, decrease asymmetry in LL (symmetry > 90°) and increase hypertrophy (asymmetry in LL circumference < 4.5 cm in distal part of the thigh) [37,38].

Once the player had recovered a large part of his mobility and neuromuscular control, sessions focused on increasing the muscle mass of the LL were then carried out.

Table 2 summarises the content worked on in each session.

The traction and mobility tests were conducted using the DynaMo digital gauge/goniometer (VALD Performance, Brisbane, Queensland, Australia). To measure force, portable uniaxial dual-force platforms (ForceDecks, FDLite, VALD Performance, Brisbane, Queensland, Australia) were utilised. Everything was connected via Bluetooth using the iOS applications DynaMo (v1.6.7) and ForceDecks (v1.9.1) on a 12.9-inch iPad Pro, 6th generation (Apple, Cupertino, CA, USA).

### 3.2. Phase 1: Postoperative (First 4 Weeks Approximately)

During this phase, the patient worked out daily at home and at the sports rehabilitation centre. Following surgery, the patient was given guidelines to prevent loss of muscle mass and recruitment, as well as to achieve full knee extension [22,23,24,46]. These postoperative instructions were as follows:

- Gain extension in the first 2 weeks. Passively, sitting or lying down with the heel supported and the leg extended and preferably raised, with nothing below the knee. Actively, same with quadriceps contractions.
- Daily use of the Game Ready system [47,48,49].
- Perform isometric contractions with the operated leg to exercise extensors, flexors, adductors and abductors, gradually increasing the difficulty.
- Strength exercises for the core, upper limbs (UL) and healthy leg (HL).
- Walking with two crutches.

During this phase, the main objectives were to reduce pain and inflammation, gain ROM (especially full extension ≤ 0°), and prevent muscle loss by improving recruitment (early awakening) of the hamstring and quadriceps muscles.

- Objective: Reduce pain (<3/10 VAS) and inflammation (between 0 and 1+), increase ROM (actively, prone position: extension 0°, flexion > 90°).

The use of the Game Ready system was essential in the first four weeks. Pressotherapy, or dynamic intermittent compression, combined with cryotherapy, helps reduce pain and therefore the need for painkillers. It also reduces inflammation, which translates into improved knee ROM [47,48,49]. The player used the Game Ready system at home for the first month, applying it for 20–40 min, 3–4 times a day, depending on the sensations.

After two weeks, diathermy therapy was another key factor in achieving a reduction in pain and inflammation, as well as an increase in ROM. Diathermy has a microcirculatory, analgesic, muscle-relaxing and tissue-healing accelerator effect, allowing the patient to progress more quickly in his rehabilitation process [50,51,52,53].

- Objective: Prevent loss of muscle mass (>4.5 cm circumference of the distal thigh) [37,38] and improve hamstring recruitment (hold gluteal and hamstring bridge in isometry for >30 s and flex 90° while standing without weight or ballast).

**Figure 4:**
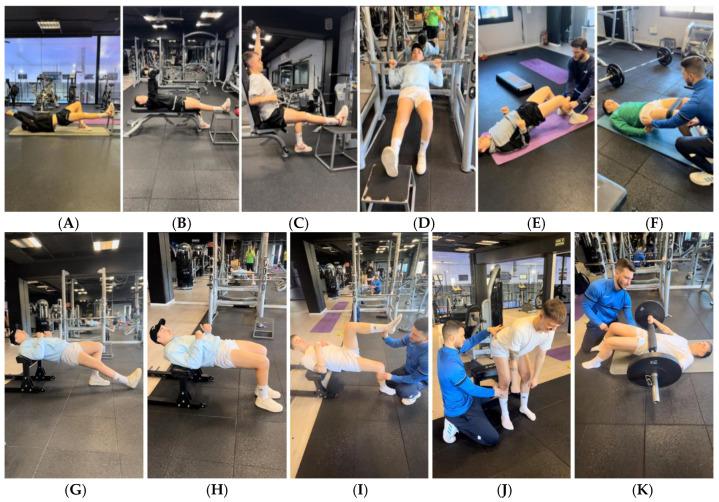
Example of exercise progression to prevent muscle loss and improve hamstring recruitment. (**A**): Asymmetric bilateral bridge in isometric position with shoulder flexion and extension to activate the core; (**B**): bench press with isometric extension of the injured leg; (**C**): isometric pull, push and extension of the injured leg; (**D**): isometric pull and extension of the injured leg; (**E**): isometric bridge with tibial fixation; (**F**): isometric bridge with tibial fixation and greater gluteus medius activation; (**G**): asymmetric hip thrust in isometric position; (**H**): symmetric hip thrust in isometric position; (**I**): unilateral hip thrust in isometric position; (**J**): deadlift technique; (**K**): weighted gluteal bridge.

Firstly, isometric exercises were chosen in CKC, with a maximum knee extension or as close as possible to 0° and with a flexion greater than 60°, with the intention of avoiding anterior displacements of the tibia and load peaks in the plasty [40,41,42,54,55,56] (Figure 4).

- Objective: Prevent loss of muscle mass (>4.5 cm circumference of the distal thigh) [37,38] and improve quadriceps recruitment (hold wall squat in isometric > 30 s and extend knee 0° in sitting position without weight or ballast).

**Figure 5:**
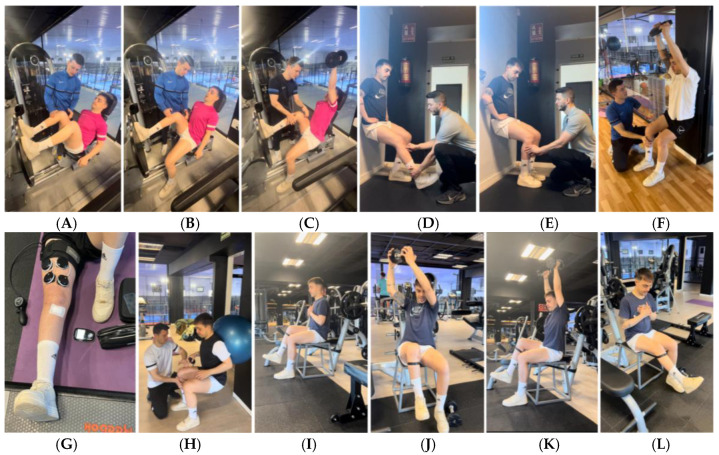
Example of exercise progression to prevent muscle loss and improve quadricep recruitment. (**A**): Isometric bilateral press push with tibial fixation; (**B**): isometric unilateral press push with tibial fixation; (**C**): isometric unilateral press push with tibial fixation and vertical push (running technique, crossed chain); (**D**): isometric wall squat with hamstring coactivation and tibial fixation; (**E**): isometric wall squat with tibial fixation; (**F**): isometric wall squat with tibial fixation and pushes in different directions; (**G**): isometric contractions with an electrostimulator in combination with blood flow restriction (BFR); (**H**): squats with BFR and tibial fixation; (**I**): isometric activation of the hip flexors with tibial fixation; (**J**): isometric activation of the hip flexors with tibial fixation and vertical thrusts with trunk rotation; (**K**): isometric activation of the hip flexors with tibial fixation and vertical thrusts; (**L**): knee extensions with proximal tibial fixation.

The same parameters as the previous objective were followed (isometric exercises in CKC, with maximum knee extension and flexion greater than 60°), except for some with proximal fixation of the tibia, as shown in Figure 5.

Initially, the player struggled to voluntarily contract his muscles, which is why sessions were started three times a week using a COMPEX SP8.0 electrostimulator (DJO France SAS, Mouguerre, France) [57,58] in combination with blood flow restriction (BFR) to improve muscle activation (Figure 5G) and also allow the patient to maintain and even gain muscle mass [22,23,24,46,57,58]. The BFR pneumatic cuff used was Occlude ApS (Aarhaus, Denmark) size L and conical shape with a circumference of 45–61 cm [59,60].

During the first few days and at the beginning of the sessions, most of the exercises applied were predominantly analytical, allowing the patient to perform them with the highest level of control possible, focusing on correct execution. Gradually, as the sense of control and activation improved, exercises involving other parts of the body and requiring greater coordination were introduced. These exercises continued to provide a safe and achievable challenge for the patient, ensuring steady and secure progress towards achieving the goals [61,62].

- Objective: Improve unaffected structures (compensatory work) and gait (walking without crutches in week 4).

Gradually, proprioception exercises and gait improvement were introduced, alongside core training and upper limb (UL) exercises [22,24,63].

The knee ligaments have a rich sensory innervation, essential for reflex responses that ensure proper joint movement. An ACL injury affects the mechanoreceptors that detect changes in movement direction, acceleration, velocity, tension and estimation of joint position. This functional instability, or loss of function, increases the risk of further injuries and impacts performance. Therefore, proprioception exercises were included to enhance joint position detection and the recognition of passive movement [63].

**Figure 6:**
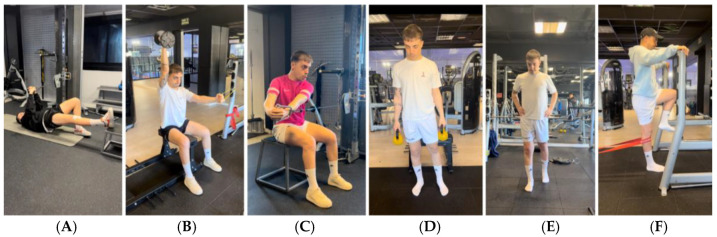
Example of exercise progression to improve unaffected structures and gait. (**A**): Asymmetric bilateral bridge in isometric with a Pallof press; (**B**): combination of a Pallof press and a vertical push; (**C**): Pallof press with rotation; (**D**): stability by shifting weight in different directions; (**E**): walking without crutches; (**F**): technical running position focusing on full knee extension, footcore activation and hip lock.

Jointly, exercises were performed in which the player shifted weight from one leg to the other in various positions and sequences to aid the development of the walking motion. Work was also carried out both with and without crutches, and even included practising running technique statically while supported on other structures. For core training, a multitude of Pallof press variations were chosen (including pushes, pulls, rotations and coordination exercises) in different positions (lying down or seated) (Figure 6).

On the other hand, after the sessions, extra strength work was carried out for UL and cardio (sitting with a battle rope) [22,24]. The contralateral leg was also worked on, known as cross-training, to help reduce problems caused by changes in the sensorimotor and musculoskeletal systems [22].

Table 3 summarises the content covered in each session.

### 3.3. Phase 2: Initial Rehabilitation—Strength-Motor Control, Low-Impact Plyometrics (Introduction of Running and Jumping Technique) (Months 2 and 3)

Once the assessment has been completed at the end of Phase 1, if the objectives set or 80% of them are achieved, Phase 2 will be carried out, consolidating and perfecting the achievements obtained in the previous phase [23,74].

This is a phase in which the main objective will be to reduce the differences between both LL, both in terms of muscle mass (asymmetry of circumference < 2 cm at proximal, middle and distal levels), as well as ROM (asymmetry < 5%), and strength (asymmetry < 20% in hamstrings and quadriceps) [22,23,24,74].

If good levels of muscular strength are recovered (symmetry 70–80%), the objectives become focused on static running technique, without displacement, both in the frontal plane and in gestures in the three dimensions of space [22,23,24,74]. In a safe and progressive way, the basic gesture is worked on, with correct body position, balance on one leg, coordination, hip lock, full extension of the knee of the supporting leg, activation of the footcore, etc. When this is under control, the next step is to work on exercises with transfer to accelerations, decelerations, changes of direction, etc. [14,22,23,24,74,75,76,77,78,79,80,81,82,83,84].

After that, and even simultaneously, low-impact plyometric work could be started [85,86,87]. Mastering jumping will be vital for subsequent running, as running involves continuously absorbing impacts while the body moves [85,86,87].

The physiotherapy treatments on a stretcher will be aimed at caring for the scar and improving the sensation of stiffness, possible pain, inflammation, etc. [88,89,90].

For compensatory work, we will continue to work on lumbopelvic stability, UL, HL and aerobic and anaerobic capacity with exercises on the stationary bike, seated battle rope or in isometric positions, swimming pool, etc. [22,91].

When the above-mentioned objectives are achieved, which is usually after approximately 3–4 months, a specific assessment is recommended [15,55,74], where the following is examined:

- Full knee extension (0°).
- Knee flexion symmetry of 95%.
- Pain < 2 on VAS scale.
- No oedema (0 or small ripples).
- Symmetry of at least 80% in quadriceps and hamstrings.
- Execution of CKC and Open Kinetic Chain (OKC) exercises with quality.

If the assessment is positive, the subject can start to run in a linear way and work on movements in different directions, braking or deceleration, changes of direction and velocity, plyometrics with medium impacts, etc., thus moving on to Phase 3 [15,74].

- Objective: To continue to promote hamstring and quadriceps muscle awakening and gains in ROM (asymmetry <5%).

**Figure 7:**
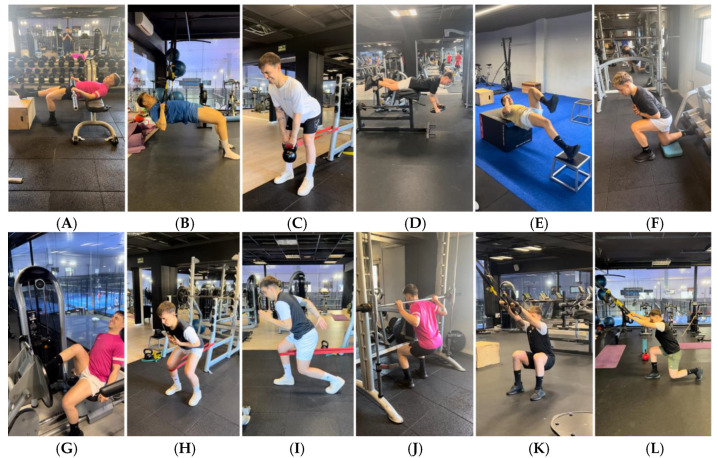
Exercises for muscle awakening of the hamstrings (**A**–**F**) and quadriceps (**G**–**L**). (**A**): Asymmetric bilateral bridge in isometrics; (**B**): isometric bridge with traction; (**C**): isometric deadlift with proximal tibial fixation; (**D**): isometric with trunk rotation (asymmetric) in Glute–Ham Developer; (**E**): unilateral isometric bridge with running technique; (**F**): unilateral isometric Nordic Curl; (**G**): unilateral isometric push-up; (**H**): isometric squat with proximal tibial fixation; (**I**): isometric lunge with proximal tibial fixation; (**J**): isometric squat on multipower; (**K**): isometric squat TRX; (**L**): isometric lunge TRX.

To achieve this, active and passive mobility exercises continued to be performed and training was carried out to a greater extent with isometrics in CKC, with maximum knee extension (0°) and with a flexion of more than 60° during the first 3 weeks of month 2 for the exercises that required a greater demand on the quadriceps, avoiding anterior displacements of the tibia and peak loads in the plasty [40,42,54,55,56]. For hamstrings, however, wider ranges (0–100°) were used in CKC and in addition to isometrics, dynamic exercises without additional weight were performed with proximal tibial fixation by the physiotherapist [40,42,54,55,56]. Few OKC exercises were performed, which were non-weight bearing and aimed at improving gait and running position (Figure 7).

- Objective: To help re-establish basic motor patterns through pool exercise.

Once the scar had closed, two sessions per week were carried out in the pool, to help re-establish patterns such as balance, walking, running and jumping [92,93]. The exercises were performed with the water at chest height and focused on motor control movements such as single-leg balance with variations, step up, etc., functional strength exercises such as squats, front and side lunges, etc., and progression exercises for running and jumping [92,93].

- Objective: To recover good levels of muscle strength (asymmetry < 20%) and anthropometric values of the thigh (asymmetry of circumference < 2 cm at distal, medial and proximal levels).

**Figure 8:**
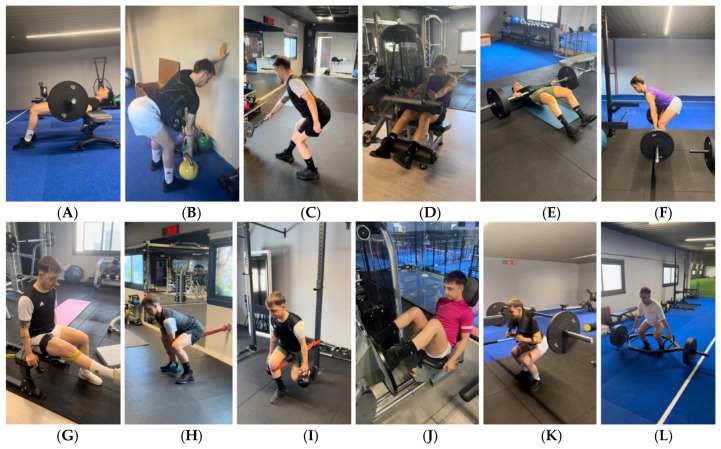
Hamstring (**A**–**F**) and quadriceps (**G**–**L**) exercises to increase anthropometric values and improve strength levels. (**A**): Hip thrust; (**B**): asymmetrical deadlift, with support; (**C**): asymmetrical deadlift on low pulley; (**D**): Lever Seated Leg Curl; (**E**): Long Lever Bridge; (**F**): Romanian deadlift; (**G**): Quadriceps Extension with BFR and resistance with elastic band attached to tibial tuberosity; (**H**): squat with elastic band attached to tibial tuberosity; (**I**): lunge with elastic band attached to tibial tuberosity; (**J**): push press; (**K**): squat with safety bar; (**L**): squat with hexagonal bar.

At month 3, all CKC exercises were performed in full range. For exercises involving quadriceps, a band fixed proximally on the anterior tuberosity of the tibia was used to reduce the risk of displacement [40,42,54,55,56]. OKC exercises were again introduced without any ballast and aimed at gait and running technique (Figure 8).

- Objective: To regain balance on one leg (motor control), improve gait and static running technique.

**Figure 9:**
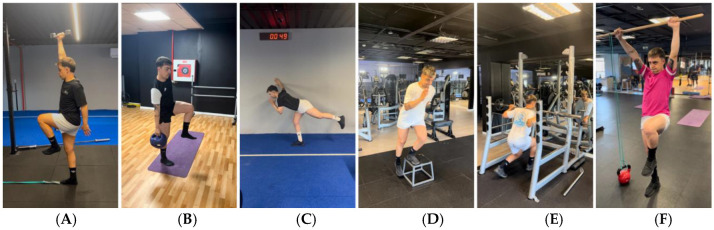
Example of exercise progression to improve balance and running technique. (**A**): Balance in running technique position with footcore activation and vertical thrust; (**B**): balance in running technique position with perturbations; (**C**): wall drill from unilateral dead lift; (**D**): step down + step up with running technique; (**E**): lunge with asymmetric loading; (**F**): balance in running technique with vertical thrust and perturbations with elastic band.

To improve balance, in addition to the pool sessions, patient worked especially on motor control through running technique exercises, with emphasis on one-leg support:

- Hip locking on the supporting leg, preventing a drop of the contralateral pelvis [75,76,77,78,79,80,81,82,83].
- Core or lumbopelvic control, stabilising and regulating the pelvis and lumbar area, while moving other parts of the body [14,71,72,84].
- Arm–leg or cross-chain coordination, requiring synchronised movement between arms and legs [64,65,66,67,68,69,70].
- The correct activation of the footcore, distributing the load well and stabilising the body by different points of contact, known as tripod [73] (Figure 9).

- Objective: To restore jumping and running patterns through plyometric work and low-impact running technique.

**Figure 10:**
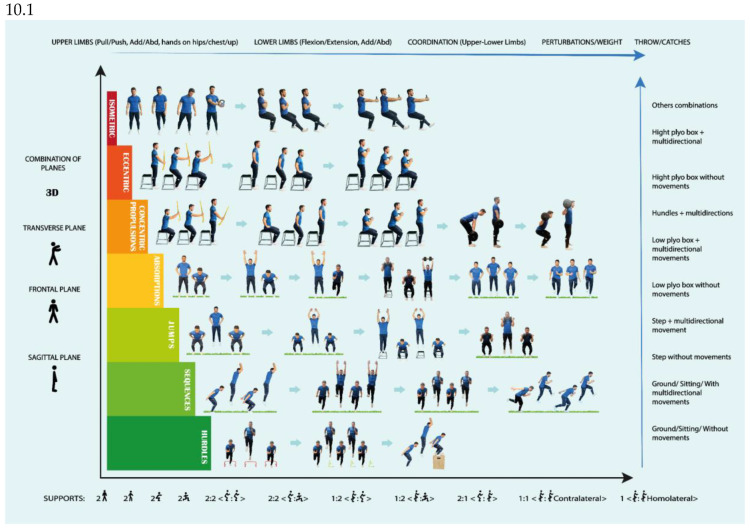

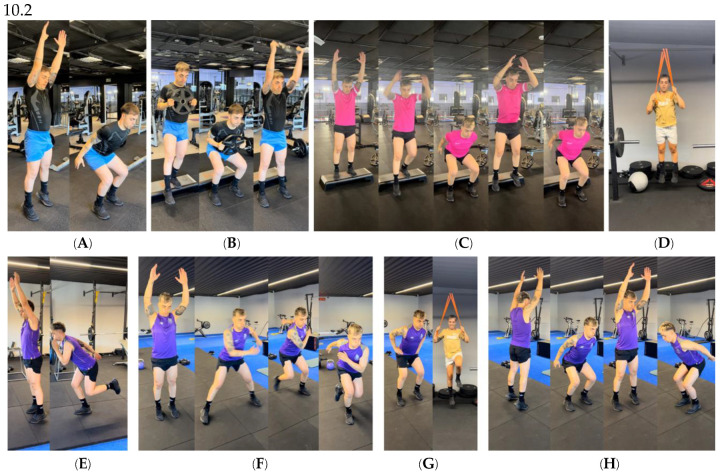
10.1: Progression carried out to re-establish the jumping pattern. 10.2: Example of exercises to work on jumping in the initial phase. (**A**): Impact absorption or deceleration gesture; (**B**): absorption + propulsion; (**C**): absorption + jump + absorption; (**D**): repeated jumps reducing impacts with an elastic band; (**E**): absorptions to one leg; (**F**): absorption to one leg + small lateral jumps; (**G**): small jumps very close together on the spot (bouncing) and repeated jumps to one leg reducing impacts with an elastic band; (**H**): deceleration or absorption gestures with simultaneous turns.

A progression was carried out based on the types of absorption and impact, types of support, different planes of space, height and length, the coordination of the different body segments and the alignment of these segments (Figure 10), avoiding knee valgus, inhibitions (drop) of the contralateral hip, hyperpronation in the stride, receiving/absorbing impact with the heels, etc. [64,65,66,67,68,69,70,85,86,87].

Specifically, the player was taught to absorb impacts (landing) in an efficient way and then worked on combining absorption and propulsion without take-off (concentric phase of the jump, which had already been worked on previously), and once controlled, the full jumping technique was introduced, ending with consecutive jumps. The progressions were mediated by the type of support (from bilateral to unilateral), the planes (sagittal to transversal and combinations), coordination (without the use of arms to throwing), height (from the ground to the maximum height of the plyometric box), length (from on the spot to as far as possible), frequency (from a single jump to several jumps in a row) and times (from the slowest to the fastest) [85,86,87].

**Figure 11:**
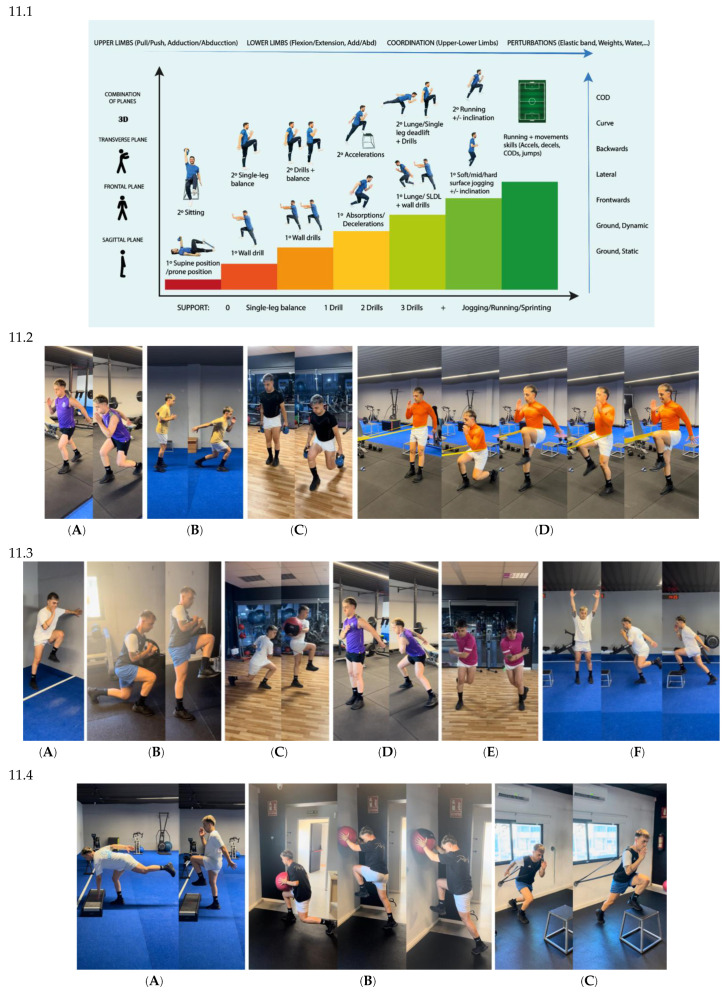

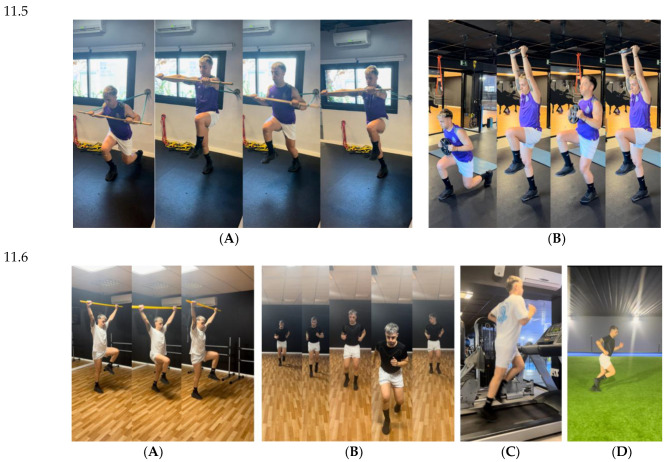
11.1: Progression carried out to re-establish the running pattern. 11.2: Example of exercises to work on the deceleration or braking gesture. (**A**): Impact absorption or deceleration gesture; (**B**): double hop + lunge absorption; (**C**): absorption by increasing the load; (**D**): deceleration or braking + running technique (drills). 11.3: Example of exercises to work on lateral displacements. (**A**): Lateral wall drill; (**B**): lateral propulsion in landmine; (**C**): lunge with running technique simulating lateral throwing (rotation); (**D**): decelerative gestures to the sides; (**E**): side to side jumps; (**F**): decelerative + change of direction + sprint gesture. 11.4: Example of exercises to work on the first steps of the sprint. (**A**): Quadruple extension + contralateral hip flexion from unilateral dead lift; (**B**): quadruple extension + contralateral hip flexion from lunge with diagonal push + drill; (**C**): quadruple extension + contralateral hip flexion from lunge with resistance. 11.5: Example of exercises to work on running steps. (**A**): Coordinated horizontal thrust with triple extension and contralateral hip flexion from lunge. The gesture is followed by a coordinated pull and a leg change (Drill) and continued with another coordinated thrust with a triple extension and contralateral hip flexion; (**B**): vertical thrust coordinated with triple extension and contralateral hip flexion from lunge. 11.6: Example of exercises to work on running technique. (**A**): Forward Drills; (**B**): forward and backward jogging, avoiding knee valgus and hip drop in the stride; (**C**): slow-speed running on treadmill; (**D**): linear running at low speed on grass.

In terms of running technique, this is something that the patient had already been internalising since the postoperative period with supine/prone exercises, sitting, and which was also worked on with balance on one leg or with the help of the wall [64,65,66,67,68,69,70,75,76,77,78,79,80,81,82,83]. In this phase, the progression goes from wall drills to straight line jogging, as can be seen in Figure 11.

Table 4 summarises the contents worked on in this phase.

### 3.4. Phase 3: Advanced Rehabilitation—Agility, Running in Different Directions, Complex Jumps and Landings (Months 4 and 5)

It begins once the objectives set in Phase 2 have been achieved, although part of the work continues to be aimed at reducing the differences between LL in muscle mass, ROM and strength [22,23,24,74].

The new objectives are aimed at improving the basic movements that have been trained in the previous phase, where the patient relearns the basic motor skills and executes them until good technique is acquired [22,23,24,25,74]. In Phase 3, this learning is consolidated in order to achieve a subsequent transfer to the specific motor skills (Phase 4), which, in this case, are the movements that the player performs on the football pitch [22,23,24,25,74].

- Objective: To continue improving strength values (asymmetry < 20%), anthropometric values of the thigh (asymmetry of circumference < 2 cm from distal to proximal areas) and ROM (knee flexion asymmetry < 5%).

Active mobility exercises were continued and training was carried out with analytical and multi-joint strength exercises in OKC and CKC in full range [22,23,24,74].

- Objective: To optimise physical conditioning and prepare the patient for the movements he will use on the pitch with exercises in the swimming pool.

Two sessions per week were performed in the pool, training medium/high intensity plyometric exercises, technical and co-ordination gestures typical of football and physical conditioning through multidirectional and variant running [92,93].

- Objective: To acquire greater skills and improve power criteria through complex jumps and running in different directions to begin high-speed exercises (HSR) and agility in the next phase.

Work continued with the progression of Figure 10, executing increasingly complex movements, increasing the height, using weights, implementing greater reactivity, turns and/or perturbations, receiving on one leg and unbalancing the player after landings, etc. [25,85,86,87].

To evaluate the improvements in this phase, different jumping tests were performed: Squat Jump, Countermovement Jump, Abalakov Jump, Drop Jump, Hop test, Single-Leg Hop Test and Single-Leg Drop Jump, although exercises such as X-Hop, Triple Hop, Timed Hop, Square Hop or Side Hop were also worked on during this phase; the main objectives were to achieve a correct technical execution, an asymmetry of less than 20% and better marks than in the previous phase [26,94,95].

For running, technique exercises were continued using all kinds of variants such as drills with disturbances (bands, weights, imbalances, etc.), braking, acceleration, turns, coordination, etc., following the progression shown in Figure 11. In Phase 2, the patient ended up jogging linearly forward. In Phase 3, the patient performed tasks such as the following [25,26,85,86,87,95,96,97,98,99]:

- Linear running, straight ahead, at a higher speed (<85% of maximum heart rate).
- Lateral running, to both sides.
- Backward running.
- Curve running (medium curve and very long curves).
- Jogging/running + braking + turn (45°, 90°, 120°, 180°).
- Changes of direction (COD): lateral push-off or side shuffles steps, lateral crossover, backwards, split-step, etc.

**Figure 12:**
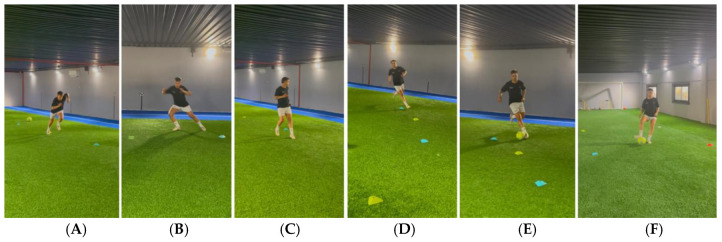

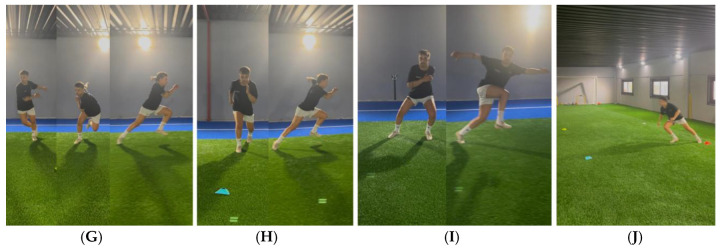
Examples of exercises for specific football skills. (**A**): Linear running; (**B**): side shuffles; (**C**): backwards running; (**D**): curve running; (**E**): ball driving; (**F**): simple passing and ball driving task; (**G**): COD via side shuffle; (**H**): COD via split-step; (**I**): COD via crossover; (**J**): COD and basic decision-making.

These tasks can be seen in Figure 12.

In the previous phase, the mechanics of sprinting, braking or change of direction were worked on before the player actually performed them through specific exercises (Figure 10 and Figure 11) [25,26,64,65,66,67,68,69,70,75,76,77,78,79,80,81,82,83,85,86,87]. In Phase 3, the patient started practising all this in the field, progressively increasing intensity, volume, repetitions, etc. [25,26,64,65,66,67,68,69,70,75,76,77,78,79,80,81,82,83,85,86,87].

Tasks were performed with a high level of control and low variability, to accumulate distances of 3–6 km at speeds of ≤65% of their maximum speed or 75% of heart rate (HR), and short-distance exercises (confined spaces of ≥4 m) with accelerations and decelerations [26].

Having worked on all the technical components (muscle cross-chains), landings or decelerations of the centre of mass (eccentric component), propulsions (concentric component), etc., it was easy for the player to put it into practice [25,26].

For the CODs, it was very important to reintroduce coordination exercises such as lateral propulsions, lateral crosses, backward and/or sideways drags, etc., which had been worked on in Phase 2 and were performed in the field with an increased intensity [25,26,96,97,98]. Initially, the CODs in this phase were performed at low speed, without fatigue, going from 45° to 180°, reducing the load on the knee joint (anterior tibial shear and subsequent ACL strain) and avoiding unwanted gestures, progressing to doing the same at medium speed [26,96,97,98].

To start the HSR and agility exercises at high intensity in the next phase, we aimed for ground contact times, in this case assessed on the strength platforms, to be >250 ms (slow stretch-shortening cycles) [26,96,97,98,99,100].

- Objective: To improve agility and decision-making in simple contexts.

At the end of this phase, once a good execution (mechanics and symmetry) of the CODs at multiple angles had been acquired, the next step was to work on agility, through COD exercises at a low-medium intensity in response to an external stimulus (external signal or instruction) [25,26,96,97,98].

- Objective: To begin with specific football training.

Once all of the above had been worked on, it was time to train with movements specific to the sport the patient was practising. The progression was as follows [25,26,99]:

- Linear movements: Linear acceleration and deceleration exercises with and without the ball (short passes of 0–10 m and low passes, heading, driving the ball and simple dribbling).
- Multidirectional movements: Accelerations and braking in different directions with and without the ball (short passes 0–10 m and low, with the head, driving the ball and simple dribbling).

### 3.5. Phase 4: Return to Training—Specific Work in the Field, Strengthening Basic and Specific Physical Skills, High-Speed and Endurance Tasks (Months 6–8)

Sessions aimed at improving strength, anthropometric parameters, running technique, jumping, etc., continued to be carried out, although what mainly characterised this phase was the incorporation of sport-specific tasks and movements to prepare the player for training with the rest of the team [15,23,24,25,26,95].

- Objective: To continue improving strength values (asymmetry < 10%), anthropometric values of the thigh (asymmetry of circumference < 2 cm in distal, middle and proximal part), ROM (asymmetry < 5%) and running and jumping technique.

Active mobility, analytical and multi-joint strength exercises continued to be performed in full range (OKC and CKC) and running and jumping technique [22,23,24,25,64,65,66,67,68,69,70,74,75,76,77,78,79,80,81,82,83,85,86,87].

- Objective: To improve endurance through the accumulation of km in tasks and speed with high-speed runs, avoiding discomfort or overtraining.

On separate days, extensive (accumulating kms with different field tasks, running without ball, HSR) and intensive (with sprints, accelerations, decelerations or COD) sessions were carried out [26].

- Objective: To improve agility, reactive strength (≤ 250 ms) and decision-making in complex, unopposed contexts.

This was followed by CODs in response to an external stimulus with visual and auditory cues [26,96,97,98]. In this phase, agility training occurs at maximum intensity and at multiple angles [25,26,96,97,98].

The player had to improve his reactivity, a prerequisite to be able to move to the next phase, since intensities >85% imply an exponential load on the tissues [26]. Therefore, jumping tests were performed with the force platforms in which the reactive force had to be ≤250 ms (fast stretch-shortening cycle) [26,100].

To improve decision-making, the external stimuli provided made the player think and presented a real challenge while trying to perform the task as fast as possible with the best possible technique. For example, variants were posed in which if he was told or pointed to a colour, he had to go the other way; mathematical calculations were also posed, etc. [61,62].

- Objective: To acquire the specific skills related to football.

As in the previous phase, work continued, this time with the following progression [25,26,99]:

- Combination of linear and multidirectional movements: With greater or lesser intensity, using the ball (10–40 m passes, 0–10 m heading, driving and dribbling) and other football-specific skills such as medium-long passes or corner crosses.
- Specific movements of 8 or 10 positions, positions in which the patient played, without opposition. Specific skills such as crossing and finishing. Exercises with the ball in fatigue and at high speed.

### 3.6. Phase 5: Return to Sport—Training with the Team (Months 9–12)

Once the player has worked on everything individually and there is no risk of relapse due to asymmetries, poor execution, inflammation or recurring pain, lack of ROM, etc., it is the ideal time to begin to train tasks with the team, focused on a future Return To Play (RTP) [15,25,26].

In this case, the player continued to improve strength, ROM, anthropometric values and technical skills in gestures such as jumping and running, but the main differentiating factor in this phase was the introduction of work with opponents [26].

At the beginning, the team performed tasks on the field and the patient had the role of ‘joker’ but helped from the outside, from a specially adapted space where he could perform tasks safely without the rest of the players being able to take the ball away from him or come into contact with him [15,25,26,61,62]. The next step was to perform tasks in the same space as the rest of the players, being a joker and without the players being able to come into contact with the patient [15,25,26,61,62]. Eventually, the patient took on the role of a joker, but opponents could steal the ball from him and come into contact with him [15,25,26,61,62].

- Objective: To improve agility, top speed, positioning and decision-making in complex contexts with opposition.

Exercises continued to be used in response to an external stimulus with visual or auditory cues or opponents (additional players) that provided increasing chaos-variability and difficulty, making them think and act with the highest possible agility-maximum speed [25,26,61,62,96,97,98].

- Objective: To acquire specific and individual skills (which characterised the player before the injury) related to football.

Gradually, the player was doing warm-ups, passing exercises, different phases of the game, etc., with the whole team [25,26]. Trained position-specific movements against one or more opponents, as well as crossing and finishing, with and without fatigue, at high speeds, etc., up to participating in real game scenarios [25,26].

Individual skills that characterised the player prior to injury were also trained and he was able to reach 95% of his pre-injury top speed, even accumulating speed volumes >85% of total speed metres/HSR [26].

### 3.7. Phase 6: RTP (From Month 12 Onwards)

The player was gradually exposed (<30, <60, <90 min) to competitive matches with the youth team and friendly matches. He continued to be monitored at all times until he performed even better than before the injury, both mentally and physically [26].

### 3.8. Criteria Used for Session Development Based on Each Phase

Preoperative Phase

During the preoperative phase, lower limb strength exercises consisted of isometric contractions lasting 20 to 40 s, with two to three sets and 1 to 2 min of rest. Additionally, concentric and eccentric contractions were organised into two to three sets per exercise and six to eight repetitions with 1–2 min rests.

Postoperative Phase

In the postoperative phase, isometric contractions of 20 to 40 s were maintained for the injured leg with the same number of sets and rests. For the healthy leg, two exercises were added with two to three sets, four to six repetitions and similar rests. Furthermore, the anterior (flexors) and posterior (extensors) crossed chains were worked with three to four exercises, two to three sets, five to eight repetitions, and 1 to 2 min of rest. Proprioception and gait training were also included in this phase with the same training structure. For improving aerobic capacity, seated battle rope training was performed, completing three sets of 15–30 s of work and 30–60 s of rest, based on sensation. Blood flow restriction (BFR) work was also combined with electrostimulation, completing a minimum of 10 contraction–relaxation cycles of the Compex SP8.0 strengthening program under the effects of BFR at 60–80% of LOP [10–20 min).

Phase 2: Initial Rehabilitation

For strength training of the injured leg in Phase 2 (initial rehabilitation), four to six exercises were performed, with two to four sets, eight to twelve repetitions, and 1 to 2 min of rest. To train the healthy leg, proprioception, gait, and cardiovascular system, exercises were maintained with the same set and repetition scheme. Motor control exercises, static running technique, jumping technique and running were added with a range of three to six exercises, two to three sets, and four to eight repetitions. Electrostimulation with BFR was increased to a minimum of 20 contraction–relaxation cycles under the Compex SP8.0 strengthening program.

Phase 3 Onward

From Phase 3 onward, exercises based on crossed chains (running technique, jumps, braking, multidirectional movements, etc.) were maintained in ranges of three to six exercises, two to three sets, and four to eight repetitions, while strength exercises transitioned to parameters of four to six exercises, with two to four sets, four to eight repetitions, and 1 to 2 min of rest.

Mobility, Activation and Core/Upper Body

For mobility, in all phases, four to six exercises of two to three sets and eight to twelve repetitions were performed. For activation and CORE/upper body work, the parameters were established as four to six exercises, two to three sets, four to eight reps, and 1–2 min of rest.

Patient Sensation

The patient’s sensations were always the reference point for adjusting the load, intensity and volume of the exercises, ensuring a safe and personalised recovery.

## 4. Discussion

The aim of the present study was to show the recovery process of a football player with a total ACL tear from the time of diagnosis to RTP, with the intention of providing a frame of reference for health care professionals working with patients who have undergone ACL surgery.

- Accelerated vs. conventional rehabilitation:

ACL surgery is increasingly common in sport and most studies or guidelines are dealing with the injury in a general way and with temporary criteria, rather than basing the process on individualisation and personal characteristics [11,14,15,16,17,18,22].

There are studies where players return to football matches 7 [14], 6 [16] and even 3 months after surgery [17]. This shows that, although there is more and more information, the criteria to be followed are very different, as are the therapies and technology used in the different cases [18].

Some studies investigate whether accelerated rehabilitation is better than conservative rehabilitation [11,15,18]. A conservative approach involves a return to sport after 9 months and has a greater emphasis on biological tissue healing [11]. It requires exercises to normalise ROM and strengthen MMII, aiming to achieve joint homeostasis, neuromuscular control and proprioception [11]. In contrast, the accelerated approach aims to return at around 6 months, including early unrestricted movement, immediate weight bearing according to tolerance and rapid removal of immobilising devices [11].

Both approaches have similar goals of restoring joint function and stability, and the evidence from some studies indicates that there is no significant difference in knee laxity at 2 years; however, the conservative approach shows a reduction in relapse rate and is beneficial for graft maturation and strength development [11].

- Influence of graft type and surgery on rehabilitation:

The maturation process of the plasty is something to take into account: the graft tissue undergoes necrosis, revascularisation and remodelling, which leads many researchers to recommend a return to full activity after 9–12 months [11,19,20]. On the other hand, the type of surgery could also mark the ideal type of rehabilitation for the patient; a surgical technique performed with a patellar tendon autograft (B-PT-B) is not the same as with a hamstring tendon (HT) [21]. In fact, the tear strength is 2376 N for B-PT-B and 4140 N for four-strand HT, compared to 2160 N for native ACL [21]. The maximum tensile strength should be compared with the maximum forces to which the reconstructed ACL is subjected in daily life (150 N), sport (400–750 N) and ‘accelerated’ rehabilitation (500 N) [21].

In line with this tensile strength, another factor to take into account would be the forces to which the plasty is subjected during the early healing/remodelling (necrosis) and proliferation/revascularisation stage, mainly leading to a correct maturation/ligamentisation phase later on [19,20]. In this regard, there has been a debate for decades on the use of exercises in CKC and/or OKC in the first months post-surgery [20,22,44,45,54,55,56,101,102,103,104,105,106,107,108,109] as inadequate rehabilitation could affect ACL laxity.

- Exercises in CKC vs. OKC:

Although it is still unclear and some studies support that there is little difference between plasties [22,109], other articles demonstrate that surgeries using ISQ may give more laxity problems versus B-PT-B [44,45,55].

This may be due to the fact that the ACL is the main brake for anterior translation of the tibia over the femur, providing 86% resistance, in addition to stopping rotations and varus/valgus angulations and providing stability [45,54,56]. If it is taken into account that the muscular action of the quadriceps in the first 0–60° of flexion results in an anterior translation of the tibia while the hamstrings (which, in the case of surgery, would be weakened) perform a posterior translation, it could be understood why HT surgery has a greater risk of laxity [44,45,56,110,111].

Thus, the lower the laxity of the knee after surgery, the lower the likelihood of progressive degeneration of cartilage, menisci and other structures [20,40,41,42]. Such laxity could be caused by OKC exercises that involve such anterior tibial translation, and thus, the plasty withstands or undergoes greater stresses [22,45,54,55,56,112,113].

- Exercise choice during the early phases:

Evidence has not determined with certainty whether OKC exercises are superior to CCC exercises in relieving pain, reducing laxity or increasing strength. However, the key may not lie in which is better, but in the manner and timing of implementation [22,110,114].

Some studies agree that performing OKC exercises the first 4 weeks post-surgery may cause laxity in the HT plasty and recommend cautious implementation from 4–6 weeks post-surgery [44,45,55,111].

Other studies support these facts and add that from week 4 onwards, it is safe to restrict the movement of OKC exercises to between 30 and 90° of flexion [22,55,112,115,116].

This corresponds with other studies showing a major role of the ACL at lower flexion angles as it receives more stress between 0–60°, while the posterior cruciate ligament receives more load from approximately 50–60° [54,56,117,118]. Peak anterior shear forces during the squat tend to occur in the first 60° of knee flexion; in CCA exercises, peak ACL loading occurs at knee angles between 10 and 30° and decreases progressively between 30 and 60° of knee flexion. Beyond 50–60° knee angles, there is minimal or no ACL loading [54,56,117,118].

In another study, it was observed that the lengths of the anteromedial and posterolateral ACL bundles were stretched more during loaded situations (box squat or lunge, CKC) than during unloaded active knee extension (OKC); in contrast, a seated knee extension, supporting 10 kg of load (OKC), involves greater lengthening and strain on AMB and PLB than during squat or lunge (CKC) [112].

Other studies also note that working in CKC has the potential to minimise stress on the reconstructed ACL, decrease anterior tibial displacement and, at the same time, normalise the physiology of the knee joint [113,116].

- Evolution in the preoperative phase:

From the beginning, consideration was given to the type of surgical technique the patient was to undergo, the injuries previously sustained, and all reports and data provided by the various clubs (including injury reports, GPS data, strength metrics, ROM measurements, etc.) were requested [18,21,22,23,24,25,26,45,54,55,56].

As already evidenced [22,23,24,25,35,55], early work both preoperatively and postoperatively favours injury recovery and shortens recovery times. Exercise was therefore the main factor to be considered from the outset. The achievement of short-term goals was in line with expectations and the patient had a progressive improvement at all times both physically and mentally [22,119].

VAS pain levels during exercise and daily activities were reduced to 1/10 at the last assessment of this phase, with instability being reported on a few occasions (Table 2). At the beginning of the phase, the player had 5/10 VAS pain when coming from home, but this was reduced to 2/10 after performing three sets of gluteal bridge isometrics [120].

Swelling decreased from 2+ to only small waves [36] and loss of mass greater than 1 cm in circumference was avoided at the proximal, medial and distal thigh [37,38].

Full ROM was achieved through passive mobility although differences in active mobility of 3° degrees in extension and 19° in flexion of the prone knee were perpetuated where the patient reported discomfort and a sensation of blockage in the popliteal fossa, possibly related to the tear of the posterior horn of the internal meniscus or the free edge of the external meniscus [22,23,24,25,40,41,42,55].

The progress in loads was adequate, indicating an improvement in muscle recruitment, since the asymmetries in the isometric push squat, squat and isometric bridge exercises were less than 7%; however, in prone knee flexion at 140° in isometry, it was 44%, where the patient continued to report the sensation of blockage in the popliteal hollow area (Table 2). The exercises assessed were performed with correct technique, avoiding knee valgus, excessive pronation or Trendelenburg sign [14,64,65,66,67,68,69,70,71,72,73,75,76,77,78,79,80,81,82,83,84].

- Evolution in the postoperative phase:

By performing the exercises proposed, pain was reduced to 1/10 VAS although presenting sensations of stiffness, swelling was reduced to 1+ and maximum extension was achieved (without forcing on a stretcher), an indispensable requirement in the first weeks (Table 3). In contrast, a knee flexion asymmetry of 10.58% remained (Table 3).

Anthropometric measurements showed a difference of 1.3 cm in the proximal area, 2.2 cm in the medial area and 1.5 cm in the distal area of the thigh (Table 3).

The exercises performed during this first month allowed the patient to maintain his recruitment capacity in isometric positions such as wall squat or gluteal and hamstring bridges, which are important for protecting the anterior drawer plasty, and even helped him to walk without crutches (Table 3). However, due to the type of surgery (HT), it was difficult to recruit concentrically the hamstrings from the first degrees of flexion (Table 3).

The main problem in this phase is that poor practice can mark the rest of the recovery (lax plasty, instability, associated meniscal problems, relapses, etc.) [20,22,40,41,42,44,45,54,55,56,112,113,119]. There may also be cases of poor or reductionist rehabilitation that do not take into account all the parameters to be improved and only focus on a few, such as mobility and strength gain, or that even place temporary criteria before individualised ones, etc. [15,22,121,122,123,124]. This is why, from this phase, we propose a work methodology aimed at improving ROM, neuromuscular recruitment, pain and other parameters, which, although safely and with limitations in certain ranges and the type of exercise, will not only allow analytical gains but also begin to improve the basis of all motor patterns and basic skills.

- Evolution of the second phase:

In Phase 2, recovery focused on improving motor control and strength work to increase muscle mass [18,21,22,23,24,25,26,45,54,55,56]. Anthropometric differences were reduced to ≤2 cm and functional tests were performed with correct technique (Table 4). In parallel, patterns such as jumping and running were reintroduced in a simple manner, with consistency based on relearning the way we move through muscle chains [14,64,65,66,67,68,69,70,71,72,73,75,76,77,78,79,80,81,82,83,84]. The clinical application of the myofascial (or muscle) chain concept is grounded in the functional and structural interconnections between muscles through connective tissue and fascia. These chains enable the efficient transmission of forces and movements throughout the body, optimising both stability and mobility. Recent studies have demonstrated that integrating exercises that respect and enhance these myofascial chains significantly contributes to neuromuscular re-education, improvement of efficient movement patterns and reduction in injury risk. In sports such as football, where technical gestures require a high degree of coordination and motor control, incorporating this approach can result in greater transfer of training and rehabilitation benefits to functional performance and injury prevention. Swimming pool training and good symmetry in ROM and strength between the LL helped the progression to be fast and effective [18,21,22,23,24,25,26,45,54,55,56,92,93]. Active and passive ROM values revealed an asymmetry ≤ 5% in all five tests and strength tests showed an asymmetry of less than 20% in all tests, except for isometric knee flexion in prone position at 140°, where the asymmetry was 48.6% and the patient described that his hamstrings would ‘cramp up’ (Table 4).

In the functional tests, asymmetry was less than 20%, except in the Squat Jump, where it was 21.9% (Table 4). Excluding the two analytical tests mentioned, the rest of the strength tests, the execution of balance exercises, walking, jumps or those aimed at running technique, showed asymmetry values suitable for starting to run in the 4th week of the 3rd month (Table 4).

Current studies and guidelines [15,18,21,22,23,24,25,26,45,54,55,56] do not address the inclusion of basic aspects of jumping, running, CODs, etc., during early stages. Instead, the focus is placed on more analytical strength or hypertrophy exercises, leading to a sudden and demanding transition in subsequent phases. This approach results in notable improvements and reduced asymmetries in analytical movements, while actions requiring greater synchronisation and coordination such as walking, running, braking, jumping or CODs display pronounced asymmetry [125,126,127,128,129,130].

Indeed, studies have demonstrated that in professional and recreational male athletes, the strength of the quadriceps and hamstrings in the unaffected limb gradually improves (increasing asymmetry) for up to six months post-ACL surgery [131]. This indicates that the strength of the unaffected limb requires constant monitoring during rehabilitation to prevent future overuse injuries [131].

An innovative progression has been implemented in this case study, incorporating strength exercises alongside the development of movement skills, such as jumping (Figure 10.1) and running (Figure 11.1) from the first month post-surgery. This progression is based on coordination criteria, muscular chain activation and technique, while also addressing ROM improvement, pain and inflammation reduction, and strength and hypertrophy asymmetries.

- Evolution in Phase 3:

The work carried out up to this point was reinforced, as reflected in the strength values, with asymmetries below 20%, asymmetries under 5% in ROM and asymmetries of less than 2 cm in the distal, middle and proximal areas of the thigh (Table 5).

The primary focus of this phase was to prepare the patient for football-related movements [23,24,25,26,99]. Linear running tests demonstrated correct technique at both low and high speeds (Table 5), maintaining normal alignment throughout the test and adapting the front-side or back-side technique depending on the speed [101,102,103,104,105,106,107,108]. It is common to observe running technique abnormalities at this stage which, if not corrected, may persist over time [104,121,125,126,127,128,129,130,131].

Additionally, considering that football is not a linear sport, training and tests were conducted for curve sprints, CODs and vertical jumps on one leg and both legs, which facilitated improvements in force production (Table 5). This prepares the player to engage in HSR exercises, agility, decision-making and football-specific contexts in Phase 4 [25,26,99].

- Evolution in Phases 4, 5, and RTP:

In Phases 4, 5, and RTP, the physical therapist–rehabilitator plays a supporting role in the activities planned by the physical trainer, working together [26,61,119,132,133]. Football-specific movements were trained, ranging from the most basic to the most specific, considering skill-agility, strength-power, endurance and actions with and without the ball performed by players in positions 8 and 10 [25,26,99,134,135].

The main objective was to ensure the player reached physical performance comparable to, or better than, their pre-injury state, guaranteeing a safe return to competition [25,26,99,136]. For this reason, tests were performed at each phase, ensuring that the patient was achieving small goals and progressing appropriately [25,26,99,136].

The importance of measuring ROM, anthropometric data, strength generated by both LL, performance quality, etc., is an essential requirement to determine whether the subject is on the right way and the appropriate physiotherapy strategy is being used. However, a large number of studies warn that upon returning to sport, athletes still present biomechanical asymmetries throughout their body, despite meeting the discharge criterion [125,126,127,128,129,130], and even the rate of new ACL injuries continues to increase [127].

In fact, horizontal jumps (distance) can achieve 97% symmetry upon returning to sport after ACL surgery, while in simple Single-Leg Vertical Jumps (height) or Single-Leg Drop Jumps, where the knee is more involved, asymmetries of 83% and 77% are found in the same subjects [126].

In other tests, such as the triple jump for distance, where symmetries can reach 97%, significant deficits in knee joint function can be masked. It has been observed that 51% symmetry is achieved during the first phase of rebound and 66% during the second [127]. Differences were more pronounced during work generation (concentric–propulsive) than during work absorption (eccentric–landing) [127].

This suggests that post-surgical biomechanics remain altered for extended periods in tasks requiring complex motor coordination [125,126,127,128,129,130], possibly due to inadequate progression in rehabilitation, not including basic skills with correct technique from the beginning, always using the same strength stimuli or not providing motor variety, etc. The injured knee tends to involve the neighbouring joints (hip, ankle, pelvis, trunk, UL) more, while the healthy knee absorbs and generates greater loads, causing compensations in the trunk and UL This asymmetry also affects muscle contraction, with increased activation of the biceps femoris and contributions from the semitendinosus, semimembranosus and soleus as protective strategies during unilateral jumps [126,128].

This highlights the need to restore not only symmetry in ground reaction forces, but also absolute performance metrics such as jump height, reactive force index, and contact times, to potentially reduce injury risk and improve overall athletic performance [125,126,127,128,129,130], as was performed in this study.

Therefore, at the end of Phases 4, 5, and 6, it would be appropriate to perform tests to assess the evolution of each parameter, for example, sprint or vertical jump tests, as previously mentioned, but also tests related to soccer, decision-making and agility (T-test, Illinois, Shuttle run, 5-cone, 505 test, Zigzag COD, Side steps, Front–side decelerations, COD with or without the ball at 45°, 90°, 120°, 180°, 360°, Crosses, kicking scenarios, etc.) [25,99,137].

Finally, while our protocol recommends a conservative RTP at 12 months postoperatively to optimise graft maturation and reduce reinjury risk, the emerging literature supports the possibility of a safe return as early as 9 months for some athletes who meet strict functional criteria. For instance, Buchheit et al. [133] reported that athletes who returned to pivoting sports before 9 months post-ACL reconstruction had a nearly sevenfold increased risk of sustaining a second ACL injury compared to those who delayed their return. Moreover, each month of delay in return to sport up to 9 months has been associated with a significant reduction in the risk of reinjury. However, athletes who demonstrate adequate quadriceps strength symmetry, pass Hop tests, and meet psychological readiness thresholds may be considered for earlier return timelines. Therefore, we advocate for individualised return-to-sport decisions guided by objective functional assessments and psychological readiness, recognizing that while a 9-month return may be feasible and appropriate for some athletes, especially those with shorter career spans, a 12-month protocol may offer enhanced long-term safety for others.

### 4.1. Limitations

The proposed case presents several limitations that merit consideration. First, this study’s findings must be interpreted within the context of a single case report, which inherently limits the generalizability of the results. Factors such as the patient’s age, pre-injury activity level, graft type used in reconstruction, and individual characteristics—including psychological readiness and comorbidities—could significantly influence rehabilitation outcomes and functional recovery. While this case illustrates the potential benefits of a tailored rehabilitation approach, it is essential to recognise that the findings cannot be directly extrapolated to all patients with ACL injuries. Therefore, further research involving larger cohorts and diverse populations is warranted to validate and refine the described protocol.

Second, this case study focuses on a player who underwent an autologous HT graft, so the progression cannot be completely extrapolated to other types of ACL surgery, although it can certainly be helpful and individualisation is necessary in all cases.

Furthermore, due to a lack of equipment, surface electromyography could not be performed to assess whether muscle recruitment improved. Instead, strength improvements in the various tests and the patient’s sensations were considered. Also, the tests performed to assess asymmetries, except for the GPS data, were not performed in a real-life game context, but rather in laboratory settings, and this could influence the results.

An additional limitation of this study is the reliance on subjective measures such as the Visual Analogue Scale (VAS) for pain assessment. While VAS is a widely accepted and practical tool in clinical settings, it is inherently subjective and may be influenced by individual perception and psychological factors. Future studies should consider integrating more objective pain assessment methods, such as pressure algometry or quantitative sensory testing, to enhance the rigor and accuracy of pain evaluation and its relationship with functional outcomes.

Finally, extended follow-up periods of 2–3 years would provide a more comprehensive assessment of the rehabilitation protocol’s effectiveness, including reinjury incidence, maintenance of physical performance and return-to-competition metrics. Future research should incorporate longitudinal tracking to strengthen the evidence base and to better inform clinical practice regarding the durability and long-term success of tailored rehabilitation protocols for ACL injuries.

### 4.2. Strengths

This study describes in detail the steps involved from preoperative to RTP, in ACL (HT) surgery, based on the current scientific literature, providing rigor and precision to the lack of information available to date.

Furthermore, it offers a perspective focused on improving movement patterns specific to the patient’s sport, working with the body holistically rather than merely analytically.

In the current context, this methodology constitutes a safe and efficient intervention strategy, facilitating the reduction in asymmetries that regrettably persist in most cases. This evidence-based and holistic approach proves to be a valuable tool in enhancing postoperative outcomes.

## 5. Conclusions

This case study provides clear and valid objective criteria that contribute to the improvement and regulation of the ACL rehabilitation process through consistent progressions, considering the muscular chains that define movement patterns in both fundamental and specific motor skills. Future research should incorporate controlled study designs, such as randomised controlled trials, to rigorously evaluate the efficacy of this tailored rehabilitation protocol and to establish clearer causal relationships between the intervention and functional recovery.

## Figures and Tables

**Table 1:** Initial patient assessment.

Tests	Results	
Anterior drawer [27,28]	Positive	Possible ACL injury
Pivot Shift [28]	Positive	Possible ACL injury
McMurray [29,30]	Positive	Possible meniscal injury
Apley [30]	Positive	Possible meniscal injury
Mobility [22]	Reduced, inflammation	
Knee extension [22]	Lack of strength/inhibition	
Knee flexion [22]	Lack of strength/inhibition	

**Table 2:** Summary of the rehabilitation process in the preoperative phase.

Sessions	Contents	Objectives	Results
Session 1	ROM assessment in active knee flexion and extension in prone position.	Assess ROM loss.	Flexion: HL 101°, IL 69°. Extension: HL 0°, IL10°.
	Dorsal ankle flexion assessment (lunge test).	Assess ROM loss.	IL: 2 cm. HL: 8.5 cm.
	Anthropometric measurement of the thighs.	Assess asymmetries.	IL: Proximal 60.1 cm, middle 54 cm, distal 43.7 cm. HL: Proximal 60.5 cm, middle 54.5 cm, distal 44 cm.
	Inflammation.	Check presence.	Yes, 2+.
	VAS (pain).	Assess pain and sensation.	5/10 at the beginning, 2/10 at the end of the session.
	Manual treatment in thigh and leg segment.	Decrease pain. Increase ROM. Improve muscle activation. Promote blood circulation.	Improves the subjective sensation of muscle tension. Improvement of ROM.
	Diathermy treatment at 500 Khz: CAP: Popliteal fossa, 5 min, P 7%, CM. RES: Thigh and leg, 8 min, P 10–20%, CM. CAP: Thigh, leg, anterolateral knee area, 10 min, P 10–25%, CM.	Improve cell regeneration. Decrease pain. Increase ROM. Improve muscle activation. Improve blood circulation.	Visible increase in blood circulation. Improved subjective sensation of muscle tension. Improved ROM.
	Exercises for knee flexors and extensors. Parameters (According to sensations): Isometric contractions of 20–40 s Sets: 2–3. Rest: 1–2 min.	Improve muscle contraction. Safely maintain strength ranges and muscle mass while avoiding pain.	Decrease in pain. Knee extension IL 6°, flexion IL 74°. Ankle dorsiflexion IL 4.5 cm. Improvement in contraction and function.
Session 2	Mobility exercises.	Reset the entire ROM.	Less stiffness.
	CORE and LL activation exercises.	Improve muscle contraction and function. Increase blood flow and temperature.	Less stiffness. Improved contraction and function.
	Isometric strength assessment of knee flexors, prone position, 140°.	Assessing muscle contractile capacity.	IL: 100 N. HL: 175 N. Asymmetry: 44%.
	LL strength work. Parameters (According to sensations): Isometric contractions of 20–40 s Sets: 2–3. Rest: 1–2 min.	Safely improve muscle contraction, strength ranges and muscle mass while avoiding soreness. Regain 90% of strength in the quadriceps and hamstrings compared to the HL.	Decreased pain. Improved contraction and function.
Sessions 3–9	Mobility exercises.	Reset the entire ROM.	Less stiffness, greater freedom of movement.
	CORE and LL activation exercises.	Improve muscle contraction and function. Increase blood flow and temperature.	Improved contraction and function.
	LL strength work. Parameters (according to sensations): Concentric and eccentric contractions. Series: 2–3. Repetitions 6–8. Rest: 1–2 min.	Safely improve muscle contraction, strength ranges and muscle mass while avoiding soreness. Regain 90% of strength in the quadriceps and hamstrings compared to the HL.	Decreased pain. Improved contraction and function.
	Diathermy (500 Khz) and manual treatment: CAP: popliteal fossa, 5 min, P 10%, CM. RES: Thigh and leg, 5 min, P 10–20%, CM. CAP: Thigh, leg 3 min, P 10–30%, CM. RES, Popliteal fossa, 3 min, P 10–15%, CM.	Improve cell regeneration. Decrease pain. Increase ROM. Improve muscle activation. Improve blood circulation.	Visible increase in blood circulation. Improved subjective sensation of muscle tension. Improved ROM.
	Inflammation.	Check for the presence of fluid.	Few, small ripples.
	VAS (pain).	Assess pain and sensations.	1/10 during daily activities and exercise. Instability rarely occurs.
	Anthropometric measurement of the thighs.	Assess asymmetries.	IL: Proximal 60.1 cm, middle 54.2 cm, distal 43.7 cm. HL: Proximal 60.5 cm, middle 54.55 cm, distal 44 cm.
	Assessment of the degrees of active mobility: 1. Knee extension, prone position. 2. Knee flexion, prone position. 3. Dorsal ankle flexion (Lunge test). Assessment of passive mobility: 4. Knee extension, supine position. 5. Knee flexion. Supine position, hip flexion 90°.	Evaluate improvement across different tests. Achieve full ROM.	1. HL: 0°, IL: 3°. 2. HL: 101°, IL: 80°. 3. HL: 8.5°, IL: 8°. 4. HL: 0° IL: 0° 5. HL: 133, IL:131°
	Strength assessment on platforms: 1. Iso push squat (3 reps of 5 s). 2. Squat (5 reps). 3. Double-leg iso bridge (3 reps of 5 s).	Evaluate asymmetries in the different tests. Regain 90% of strength in the quadriceps and hamstrings compared to the HL.	1. 2032 N (HL produces 3.6% more force) 2. 1410 N (HL produces 6.5% more force) 3. 489 N (HL produces 6.5% more force)
	Assessment of the force exerted with the tensile gauge: 1. Knee flexion in prone position at 140° in isometry (3 sets of 5 s).	Evaluate LL asymmetries. Regain 90% of strength in the quadriceps and hamstrings compared to the HL.	HL: 179 N IL: 100 N Asymmetry: 44%

CAP: capacitive; CM: continuous mode; HL: healthy leg; IL: injured leg; LL: lower limb; N: newtons; P: power; RES: resistive; ROM: range of motion.

**Table 3:** Summary of the rehabilitation process in the postoperative phase.

Sessions	Contents	Objectives	Results
Week 1 (home, unsupervised, following instructions)	Game Ready system: 20–40 min, 3–4 times a day.	Reduce pain. Decrease inflammation by promoting drainage. Increase ROM.	Elimination of medication. Significant reduction in pain and inflammation. Daily improvement of ROM.
	Passive–active knee extension (at home, lying down or sitting with the help of furniture).	Gain full extension.	Improved ROM. Feeling of freedom and greater ability to extend the knee.
	Passive–active knee flexion (at home, lying down or sitting with the help of furniture) without forcing.	Gain flexion.	Improved ROM. Feeling of freedom and greater ability to flex the knee.
	Knee flexor/extensor exercises. Parameters (depending on sensations): Isometric contractions for 20–40 s. Sets: 2–3. Rest 1–2 min.	Improve muscle contraction. Safely maintain strength ranges and muscle mass while avoiding pain.	Decreased pain. Increased ROM. Feeling of weakness and lack of strength. However, improved contraction capacity for days (subjective).
Week 2 (home and gym)	Game Ready system: 20–40 min, 3–4 times a day.	Reduce pain. Decrease inflammation by promoting drainage. Increase ROM.	Elimination of medication. Significant reduction in pain and inflammation. Daily improvement of ROM.
	Active ROM assessment in prone knee flexion–extension.	Assess ROM loss. Reach full extension.	Flexion: HL 101°; IL 45° Extension: HL 0°; IL 4°
	Anthropometric measurement of the thighs.	Assess for loss of muscle mass.	IL: Proximal 58.8 cm, middle 52 cm, distal 42.3 cm. HL: Proximal 60.1 cm, middle 54.2 cm, distal 43.8 cm.
	Inflammation.	Assess for presence of fluid. Be at 0–1+ after the first month of rehabilitation.	Yes. 2+
	VAS (pain)	Assess pain and sensation.	3/10. Stiffness (popliteal fossa)
	Diathermy (500 kHz) and manual therapy: CAP: Popliteal fossa, 5 min, P 10%, CM. RES: Thigh and leg, 5 min, P 10–20%, CM. CAP: Thigh and leg, 3 min, P 10–30%, CM. RES, Popliteal fossa, 3 min, P 10–15%, CM. CAP: Lower back, 2 min, P 30%, CM. RES: Lower back, 2 min, P 20%, CM.	Improve cell regeneration. Decrease pain. Increase ROM. Improve muscle activation. Promote circulation.	Acceleration of the recovery process. Visible increase in blood circulation. Improvement of the subjective sensation of muscular tension. Improved ROM.
	Active mobility assessment and exercises.	Achieve maximum knee extension (0°). Improve knee flexion.	Extension: HL 0°; IL 2° D. prono. Flexion: HL 105°; IL 82° D. prono.
	Exercises to improve knee flexor and extensor contraction (BFR Occlude, Compex SP8.0).	Achieve activation of the flexor and extensor musculature. Complete a minimum of 10 contraction–relaxation cycles of the Compex SP8.0 strengthening program under the effects of the BFR (10–20 min). Obtain the secondary benefits that the BFR provides.	Reduction in pain. Improvement of blood circulation. Improved sensation of mobility. Improved sensation of contraction.
	Isometric and/or muscle chain strength exercises * [64,65,66,67,68,69,70,71,72,73] for IL: 3–4 exercises, 2–3 sets, 5–8 reps, 1–2 for rests (all based on sensations).	Improve recruitment and maintain muscle mass, especially in hamstrings and quadriceps. Encourage transfer to gestures (walking/running) by including other body segments.	Decreased pain. Improved contraction and function.
	Proprioception and gait: 3–4 exercises, 2–3 sets, 4–6 reps, 1–2 min for rests (all according to feeling).	Improve stability and body awareness. Achieve walking without crutches in 1 month.	Increased knee awareness (touch). Standing without crutches for 30 s. Walking with crutches while removing weight.
	HL strength work (at home or unsupervised gym): 2 exercises, 2–3 sets, 4–6 reps, 1–2 min rest (all according to feeling).	Achieve strength gains in the untrained counterpart muscles of the contralateral LL. Maintain/gain strength in HL.	Feeling of greater control with HL.
	CORE and UL exercises (at home or individualised but unsupervised gym): 4–6 exercises, 2–3 sets, 4–8 reps, 1–2 min rest (all according to sensations).	Improve stability and transfer of strength from the lumbo-pelvic region to other regions (UL, LL). Improve strength in UL.	Good adaptation to the load. Reduction in postoperative pain (compensation, couch, etc.) in the lumbar area.
	Cardio: seated battle rope (At home or individualised gym but not supervised); 3 sets of 15–30 s work and 30–60 s rest, according to sensations.	Recover lost aerobic and anaerobic capacity.	Less feeling of tiredness after several sessions.
Week 3 (home and gym)	Game Ready system: 20–40 min, 3–4 times a day.	Reduce pain. Decrease inflammation by promoting drainage. Increase ROM.	Elimination of medication. Pain 2/10, less inflammation (1+). Daily improvement of ROM.
	Exercises and active mobility assessment.	Restore full knee extension (0°). Improve knee flexion. Reach 90° prone position.	Extension: HL 0° IL 0°. Flexion: HL 105°; IL 92°.
	Exercises to improve knee flexor and extensor contraction (BFR Occlude, Compex SP8.0).	Achieve activation of the flexor and extensor musculature. Complete a minimum of 10 contraction–relaxation cycles of the Compex SP8.0 strengthening programme under the effects of the BFR (10–20 min). Obtain the secondary benefits that the BFR provides.	Reduction in pain. Improvement of blood circulation. Improved sensation of mobility. Improved sensation of contraction.
	Isometric and/or muscle chain strength exercises for IL: 3–4 exercises, 2–3 sets, 5–8 reps, 1–2 min rests (all according to sensations).	Improve recruitment and maintain muscle mass, especially in the hamstrings and quadriceps. Encourage transfer to gestures (walking/running) by including other body segments.	Decreased pain. Improved contraction and function.
	Proprioception and gait: 3–4 exercises, 2–3 sets, 4–6 reps, 1–2 min rests (all according to sensations).	Improve stability and body awareness. Achieve walking without crutches in 1 month.	Awareness: knee, hip and footcore. Standing without crutches > 30 s. Slow walking without crutches.
	Diathermy at 500 Khz: CAP: popliteal fossa, 5 min, P 15%, CM. RES: Thigh and leg, 5 min, P 10–25%, CM. CAP: Thigh, leg 3 min, P 10–40%, CM. RES, Popliteal fossa, 3 min, P 10–15%, CM. CAP: Lower back, 2 min P 35%, CM. RES: Lower back, 2 min P 25%, CM.	Improve cell regeneration. Decrease pain. Increase ROM. Improve muscle activation. Improve circulation.	Acceleration of the recovery process. Visible increase in blood circulation. Improvement of the subjective sensation of muscular tension. Improved ROM.
	HL strength work (at home or gym individualised but not supervised): 2 exercises, 2–3 sets, 4–6 reps, 1–2 min rest (all according to sensations).	Achieve strength gains in the untrained counterpart muscles of the contralateral LL. Maintain/gain strength in HL.	Load increase with HL.
	CORE and UL exercises (at home or individualised but not supervised gym): 4–6 exercises, 2–3 sets, 4–8 reps, 1–2 min rest (all according to feelings).	Improve stability and transfer of strength from the lumbo-pelvic region to other regions (UL, LL). Improve strength in UL.	Increased load. Disappearance of postoperative pain (compensation, couch, etc.) in the lumbar area.
	Cardio: seated battle rope (at home or individualised gym but not supervised); 3 sets of 15–30 s work and 30–60 s rest, according to sensations.	Recover lost aerobic and anaerobic capacity.	Less feeling of tiredness after several sessions.
Week 4 (home and gym)	Game Ready system: 20–40 min, 3–4 times a day.	Reduce pain. Decrease inflammation by promoting drainage. Increase ROM.	Elimination of medication. No pain, inflammation (1+). Daily improvement of ROM.
	Exercises and active mobility assessment.	Maintain full extension (0°). Reach 90° of knee flexion while lying prone.	Extension: HL 0° IL 0° Flexion: HL 104° IL 93°
	VAS (pain).	Assess pain and sensation.	1/10. Less stiffness sensation.
	Inflammation assessment.	Assess the presence of fluid. Be at 0–1+	Yes, 1+
	Isometric assessment: 1. Isometric glute bridge (30 s). 2. Isometric hamstring bridge (30 s). 3. Isometric wall squat (30 s). 4. Active knee flexion without weight. 5. Active knee extension without weight.	Assess recovery of strength post-surgery. Assess active ROM.	1. Ok. 2. Ok. 3. Ok. 4. Lack of activation, feeling of muscle cramp. 5. Ok.
	Gait assessment.	Assess gait and possible compensations.	No crutches, OK.
	Exercises to improve knee muscles recruitment (flexors and extensors). (BFR Occlude, Compex SP8.0).	Achieve activation of the flexor and extensor musculature. Complete a minimum of 10 contraction–relaxation cycles of the Compex SP8.0 strengthening programme under the effects of the BFR (10–20 min). Obtain the secondary benefits that the BFR provides.	Reduction in pain. Improvement of blood circulation. Improved sensation of mobility. Improved sensation of contraction.
	Isometric and/or muscle chain strength exercises for IL: 3–4 exercises, 2–3 sets, 5–8 reps, 1–2 min rests (all according to sensations).	Improve recruitment and maintain muscle mass, especially in the hamstrings and quadriceps. Encourage transfer to gestures (walking/running) by including other body segments.	Decreased pain. Improved contraction and function.
	Proprioception and gait: 3–4 exercises, 2–3 sets, 4–6 reps, 1–2 min rests (all according to sensations).	Improve stability and body awareness. Achieve walking without crutches in 1 month.	Awareness: knee, hip and footcore. Standing without crutches > 30 s. Slow walking without crutches.
	Diathermy at 500 Khz: CAP: popliteal fossa, 5 min, P 20–35%, CM. RES: Thigh and leg, 5 min, P 20–30%, CM. CAP: Thigh, leg 3 min, P 20–45%, CM. RES: Popliteal fossa, 3 min, P 20–30%, CM. CAP: Lower back, 2 min P 35%, CM. RES: Lower back, 2 min P 30%, CM.	Improve cell regeneration. Decrease pain. Increase ROM. Improve muscle activation. Improve circulation.	Acceleration of the recovery process. Visible increase in blood circulation. Improvement of the subjective sensation of muscular tension. Improved ROM.
	HL strength work (at home or gym individualised but not supervised): 2 exercises, 2–3 sets, 4–6 reps, 1–2 min rest (all according to sensations).	Achieve strength gains in the untrained counterpart muscles of the contralateral LL. Maintain/gain strength in HL.	Load increase with HL.
	CORE and UL exercises (at home or individualised but not supervised gym): 4–6 exercises, 2–3 sets, 4–8 reps, 1–2 min rest (all according to feelings).	Improve stability and transfer of strength from the lumbo-pelvic region to other regions (UL, LL). Improve strength in UL.	Increased load. Elimination of postoperative pain (compensation, couch, etc.) in the lumbar area.
	Cardio: seated battle rope (at home or individualised gym but not supervised); 3 sets of 15–30 s work and 30–60 s rest, according to sensations.	Recover lost aerobic and anaerobic capacity.	Greater capacity to perform the exercise more quickly. Faster recovery sensation.

* Muscle chain or myofascial chain training is based on training the body according to how it moves and the relationship/connection between the different muscles and fasciae. For example, when a player run, we can observe that, very briefly, there is an anterior chain acting and a posterior chain acting. When an LL goes forwards, the opposite UL also goes forward, while on the other hand, a UL goes backwards, and the opposite LL is also behind. BFR: blood flow restriction; CAP: capacitive; CM: continuous mode; HL: healthy leg; IL: injured leg; LL: lower limbs; P: power; RES: resistive; ROM: range of motion; UL: upper limbs.

**Table 4:** Summary of the rehabilitation process in Phase 2 (Strength/motor control, jumping and low-impact running technique).

Period	Contents	Objectives	Results
Month 2, weeks 1–3 (pool and gym).	Diathermy at 500 Khz: CAP: Popliteal fossa, 5 min, P 40–50%, CM. RES: Thigh and leg, 5 min, P 30–40%, CM. CAP: Thigh and leg 3 min, P 35–55%, CM. RES: Popliteal fossa, 3 min, P 30–40%, CM. CAP: Lower back, 2 min P 35–45%, CM. RES: Lower back, 2 min P 30–40%, CM.	Improve cell regeneration. Decrease pain. Increase ROM. Improve muscle activation. Improve circulation.	Acceleration of the recovery process. Visible increase in blood circulation. Improvement of the subjective sensation of muscular tension. Improved ROM.
	Exercises to improve knee muscles recruitment (flexors and extensors). (BFR Occlude, Compex SP8.0).	Achieve activation of the flexor and extensor musculature. Complete a minimum of 10 contraction–relaxation cycles of the Compex SP8.0 strengthening programme under the effects of the BFR (10–20 min). Obtain the secondary benefits that the BFR provides.	Reduction in pain. Improvement of blood circulation. Improved sensation of mobility. Improved sensation of contraction.
	Isometric and/or muscle chain strength exercises for IL: 3–4 exercises, 2–3 sets, 5–8 reps, 1–2 min rests (all according to sensations).	Improve recruitment and maintain muscle mass, especially in the hamstrings and quadriceps. Encourage transfer to gestures (walking/running) by including other body segments.	Decreased pain. Improved contraction and function.
	Proprioception and gait: 3–4 exercises, 2–3 sets, 4–6 reps, 1–2 min rests (all according to sensations).	Improve stability and body awareness. Multidirectional walking without crutches.	Increased knee awareness. Standing and walking without crutches.
	HL strength work (at home or gym individualised but not supervised): 2 exercises, 2–3 sets, 4–6 reps, 1–2 min rest (all according to sensations).	Achieve strength gains in the untrained counterpart muscles of the contralateral LL. Maintain/gain strength in HL.	Load increase with HL.
	CORE and UL exercises (at home or individualised but not supervised gym): 4–6 exercises, 2–3 sets, 4–8 reps, 1–2 min rest (all according to feelings).	Improve stability and transfer of strength from the lumbo-pelvic region to other regions (UL, LL). Improve strength in UL.	Increased load. Elimination of postoperative pain (compensation, couch, etc.) in the lumbar area.
	Cardio: seated battle rope (at home or individualised gym but not supervised); 3 sets of 15–30 s work and 30–60 s rest, according to sensations.	Recover lost aerobic and anaerobic capacity.	Greater capacity to perform the exercise more quickly. Faster recovery sensation.
	Pool exercises (individualised, unsupervised): 1 h twice a week.	Improve motor control. Perform functional strength exercises that allow you to progress to later work on jumping and running.	Increased sense of control and strength. Less fear when performing more complex tasks.
	Exercises and active mobility assessment.	Maintain full extension (0°). Reach 100° of knee flexion while lying prone.	Extension: HL 0° IL 0° Flexion: HL 105° IL 102°
	Inflammation assessment.	Assess for presence of fluid. Be at 0–1+.	Yes. 1+
	VAS pain.	Assess pain and sensation.	0/10. Feeling knee emptiness.
	Anthropometric measurement of the thighs.	Assess for loss of muscle mass.	IL: Proximal 59 cm, middle 52.8 cm, distal 42.7 cm. HL: Proximal 60.4 cm, middle 54.6 cm, distal 44 cm.
	Isometric tests: 1. Hold isometric glute bridge with IL. 2. Hold isometric hamstring bridge with IL. 3. Iso push barbell squat 4. Active knee flexion without weight. 5. Active knee extension without weight.	Evaluate strength recovery post-surgery. Evaluate active ROM. Identify the % asymmetry between IL and HL.	1. Ok. 2. Ok. 3. 1.752 N (HL produces 27,3% more force) 4. Ok. 5. Ok.
Month 2, week 4 and month 3 complete (pool and gym). Assessment conducted in the last week of month 3.	Diathermy at 500 Khz: CAP: Popliteal fossa, 5 min, P 40–50%, CM. RES: Thigh and leg, 5 min, P 30–40%, CM. CAP: Thigh and leg, 3 min, P 35–55%, CM. RES: Popliteal fossa, 3 min, P 30–40%, CM. CAP: Lower back, 2 min P 35–45%, CM. RES: Lower back, 2 min P 30–40%, CM.	Improve cell regeneration. Decrease pain. Increase ROM. Improve muscle activation. Improve circulation.	Acceleration of the recovery process. Visible increase in blood circulation. Improvement of the subjective sensation of muscular tension. Improved ROM.
	Strength exercises to reduce asymmetry in strength and muscle mass: 4–6 exercises, 2–4 sets, 8–12 reps, 1–2 min. rests (all based on sensations).	Reduce strength asymmetries (<20%) and muscle mass (<2 cm). Improve muscle contractile function.	Increased ROM, contractile capacity, confidence and stability. Decreased sensation of discomfort and pain.
	Motor control and static running technique exercises: 3–6 exercises, 2–3 sets, 5–8 reps, 1–2 min. rests (all based on sensations).	Restore neuromuscular control, avoiding compensations and improving proprioception and stability. Promote transfer to movements (walking/running) by involving all body segments.	Improved contraction, function, and confidence. Awareness of knee, hip, and footcore. Balance on one leg for >30 s. Multidirectional gait without crutches.
	Jumping technique exercises: 3–6 exercises, 2–3 sets, 4–6 reps, 1–2 min. rests (all based on sensations).	Re-establish the jumping motor pattern. Ensure good jumping technique (LL-UL coordination, impact absorption, propulsion, etc.) that will allow for good future performance and prevent relapses.	Ability to perform pain-free and fear-free absorptions. Increasingly symmetrical propulsions. Small jumps combining planes and heights with correct technique, avoiding unwanted compensations and misalignments.
	Running technique exercises: 3–6 exercises, 2–3 sets, 4–6 reps, 1–2 min. rests (all based on sensations).	Re-establish the running motor pattern. Ensure good running technique (LL-UL coordination, impact absorption, quadruple extension, hip lock, etc.) that will allow for good future performance and prevent relapses.	Ability to move the body at a linear “jog” pace (frontal, lateral, and backward) with confidence. Execution of drills with good technique (correct UL-LL coordination, correct posterior chain extension, no hip drop, no knee valgus, etc.)
	HL strength work (at home or gym individualised but not supervised): 2 exercises, 2–3 sets, 4–6 reps, 1–2 min rest (all according to sensations).	Achieve strength gains in the untrained counterpart muscles of the contralateral LL. Maintain/gain strength in HL.	Load increase with HL.
	CORE and UL exercises (at home or individualised but not supervised gym): 4–6 exercises, 2–3 sets, 4–8 reps, 1–2 min rest (all according to feelings).	Improve stability and transfer of strength from the lumbo-pelvic region to other regions (UL, LL). Improve strength in UL.	Increased load. Disappearance of postoperative pain (compensation, couch, etc.) in the lumbar area.
	Cardio: seated battle rope (at home or individualised gym but not supervised); 3 sets of 15–30 s work and 30–60 s rest, according to sensations.	Recover lost aerobic and anaerobic capacity.	Less feeling of tiredness after several sessions.
	Active mobility assessment and exercises.	Maintain full extension (0°). Maintain 100% knee flexion in the prone position.	Extension: HL 0° IL 0°. Flexion: HL 104° IL 102°.
	VAS pain.	Confirm there is no pain so the patient can run and progress to the next phase.	0–2 depending on the gesture/impact. Not described as pain, but rather as a sensation of something missing.
	Inflammation assessment.	Evaluate for fluid. Be at 0–1+.	Between 0 and 1 (Small wave on the medial side with a downward stroke).
	Anthropometric measurement of the thighs.	Assess for loss of muscle mass.	IL: Proximal 59 cm, middle 53.1 cm, distal 42.8 cm. HL: Proximal 60.5 cm, middle 55.1 cm, distal 44.2 cm.
	Gait assessment.	Evaluate gait and possible compensations.	Ability to walk in all directions without compensating.
	Active range of motion assessment: 1. Knee extension, prone position. 2. Knee flexion, prone position. 3. Ankle dorsiflexion (Lunge test). Passive range of motion assessment: 4. Knee extension, supine position. 5. Knee flexion, supine position, hip flexion 90°.	Evaluate improvement in the different tests. Achieve full ROM.	1. HL: 0°, IL: 0°. 2. HL: 105°, IL: 103°. 3. HL: 8.5°, IL: 8,3°. 4. HL: 0° IL: 0° 5. HL: 134, IL:129°
	Strength assessment on platforms: 1. Iso push squat (3 repetitions of 5 s). 2. Squat (5 repetitions). 3. Double-leg iso bridge (3 repetitions of 5 s).	Evaluate asymmetry in the various tests. Regain 80% strength in the quadriceps and hamstrings compared to the healthy side.	1. 1858 N (HL produces 18.6% more force) 2. 1219 N (HL produces 17.5% more force) 3. 489 N (HL produces 19.7% more force)
	Strength assessment with a pull gauge: Prone knee flexion at 140° in isometric position (3 repetitions of 5 s).	Evaluate asymmetry between both legs. Regain 80% strength in the hamstrings compared to the healthy side.	HL: 152 N IL: 78 N 48.6% asymmetry. In this test, the patient described feeling like his hamstrings were going to cramp and he could not exert any more force.
	Test funcionales 1. Maintain single-leg balance for 30 s in running technique position. 2. Perform a single-leg squat starting from a seated position at 90°. 3. Calf raises. 4. SJ. 5. CMJ. 6. Abalakov Jump. 7. Drop Jump (30 cm). 8. Hop test. 9. Advanced Drills. Functional Tests: 1. Hold one-legged balance for 30 s in a running technique position. 2. Perform a single-leg squat from a seated position at 90°. 3. Calf raises. 4. SJ. 5. CMJ. 6. Abalakov jump. 7. Drop Jump (30 cm high). 8. Hop test. 9. Drills moving forward.	Assess whether progress has been adequate. Check whether the patient can move on to the next phase.	1. Ok 2. Ok, correct alignment. 3. Ok 4. 19.5 cm, Peak Landing Force—Bilateral 3.833 N, 21.9% asymmetry with the left, RSI-modified 0.58 ms. 5. 27.2 cm, Peak Landing Force—Bilateral 2.819 N, 16.5% asymmetry with the left, RSI-modified 0.34 ms. 6. 30.7 cm, Peak Landing Force—Bilateral 2.776 N, 19.7% asymmetry with the left, RSI-modified 0.36 ms. 7. 34.3 cm, Peak Drive-Off Force—Bilateral 1.949 N, 10.7% 10.7% asymmetry (right produces more force), RSI (Fligth Time/Contact time) 0.80 ms. 8. Mean Jump Heigh (Flight Time) 14.4 cm, Mean RSI (Jump Height/Contact Time 0.91 ms, Mean Peak Force—Bilateral 4.713 N (asymmetry 7,9% left), n° Hops/reps 6. 9. Ok
	Conclusion after a full 3-month assessment (4th week of the 3rd month)	Optimal results for the patient to start running.	

BFR: blood flow restriction; CAP: capacitive; CMJ: Countermovement Jump; HL: healthy leg; IL: injured leg; LL: lower limbs; MC: continuous mode; P: power; RES: resistive; SJ: Squat Jump; ROM: range of motion; RSI: Reactive Strength Index; UL: upper limbs; VAS: Visual Analogue Scale.

**Table 5:** Assessment performed in Phase 3. Agility, running in different directions, jumps and complex landings (months 4 and 5).

Period	Contents	Objectives	Results
Last week of month 5.	VAS pain.	Evaluate pain to progress to the next phase.	0/10.
	Inflammation assessment.	Evaluate for fluid presence. Be at 0–1+.	0.
	Anthropometric measurement of the thighs.	Restore lost muscle mass.	IL: Proximal 59.3 cm, middle 53.3 cm, distal 43 cm. HL: Proximal 60.5 cm, middle 55.2 cm, distal 44.3 cm.
	Treadmill running assessment, without inclination [109,110,111,112,113,114,115,116].	Identify compensations when running on a treadmill at low, medium and high speeds. Improve running technique in each phase (flight, support, speeds, etc.).	Medium stance. No lateral trunk tilt. Hip: Normal axis (no pelvic drop). Knee: Normal axis (no valgus/varus). Ankle: Normal axis (no excessive pronation or supination). Slow running (6 km/h): backside, impact absorption with rearfoot. Running at 15 km/h: Frontside, impact absorption with forefoot.
	20 m linear sprint assessment.	Evaluate total time and the difference between the sprint start, first meters and last meters.	Total time: 3.348 s Time in first 5 m: 1.182 s Time in second 5 m: 0.797 s Time in last 10 m: 1.369 s
	Non-linear sprint assessment: 1. 15 m Curved Sprint (both sides). 2. 180° COD, 15 m front direction and 15 m opposite (both sides).	Evaluate the difference between left and right sides.	1. Starting to the right: 2.23 s Starting to the left: 2.18 s 2. Turning to the right: 6.22 s Turning to the left: 6.04 s
	Strength assessment in platforms: 1. Squats (5 repetitions). 2. Deadlift (5 repetitions). 3. Single-Leg Squat.	Evaluate asymmetry in the different tests. Recover at least 80% of strength in the quadriceps and hamstrings compared to the healthy side.	1. 2378 N (HL produces 3.8% more strength) 2. 1373 N (IL produces 9.5% more strength) 3. Peak Force—Left (853 N), right (870 N), asymmetry 1.9%, Maximum Negative Displacement—Left (22.2 cm), Right (21.0 cm), asymmety = 5.41%, Concentric Peak Power/BM—Left (5.50 W/Kg), Right (5.92 W/Kg), asymmetry = 7%.
	Assessing strength exerted with a gauge.	Evaluate improvement after the previous test. Recover at least 80% of strength in the hamstrings compared to the healthy side.	HL: 229 N. IL: 184.9 N. 19.2% of asymmetry Still feeling cramp sensations.
	Functional test (Jump): 1. SJ. 2. CMJ. 3. Abalakov Jump. 4. Drop Jump (30 cm high). 5. Hop test. 6. Single Hop test. 7. Single-Leg Drop Jump (30 cm high).	Assess whether there are asymmetries greater than 20%. Check if the patient can move on to the next phase.	1. 28.8 cm, Peak Landing Force—Bilateral 3.232 N, 19.3% of asymmetry with left, RSI-modified 0.83 ms. 2. 30.4 cm, Peak Landing Force—Bilateral 2.818 N, 16.8% of asymmetry with right, RSI-modified 0.42 ms. 3. 33.0 cm, Peak Landing Force—Bilateral 2.157 N, 13.3% of asymmetry with right, RSI-modified 0.42 ms. 4. 34.6 cm, Peak Drive-Off Force—Bilateral 1.892 N, 2% of asymmetry with (right produces more strength), RSI (Fligth Time/Contact time) 0.73 ms. 5. Mean Jump Heigh (Flight Time) 15.3 cm, Mean RSI (Jump Height/Contact Time 0.92 ms, Mean Peak Force—Bilateral 5.013 N (asymmetry 9.3% rigth), n° Hops/reps 6. 6. Mean Jump Heigh (Flight Time) left (11.8 cm) right (9.9 cm), asymmetry = 16.1%, Mean RSI (Jump Height/Contact Time left (0.46 ms) right (0.39 ms), asymmetry = 15.2% Mean Peak Force—left (2.580 N) right (2.165 N), asymmetry = 16.1%, n° Hops/reps 5. 7. Jump Height (Imp-Mom)—Left Side (4.1 cm), Peak Drive-Off Force 1.539 N, RSI (JH (Flight Time)/Contact Time) 0.18 ms. Jump Height (Imp-Mom)—Right Side (2.6 cm), Peak Drive-Off Force 1.508 N, RSI (JH (Flight Time)/Contact Time) 0.29 ms.
	Conclusion after a full 5-month assessment (4th week of the 5th month)	Optimal results for the patient to begin Phase 4, RETURN TO TRAINING.	

CMJ: Countermovement Jump; HL: healthy leg; IL: injured leg; IMP: impulse; JH: jump height; Mom: momentum; RSI: Reactive Strength Index; SJ: Squat Jump; VAS: Visual Analogue Scale.

## Data Availability

The data presented in this study are available on request from the corresponding author. The data are not publicly available due to privacy restrictions.
